# Genome Analysis of *Lagocephalus sceleratus*: Unraveling the Genomic Landscape of a Successful Invader

**DOI:** 10.3389/fgene.2021.790850

**Published:** 2021-12-08

**Authors:** Theodoros Danis, Vasileios Papadogiannis, Alexandros Tsakogiannis, Jon B. Kristoffersen, Daniel Golani, Dimitris Tsaparis, Aspasia Sterioti, Panagiotis Kasapidis, Georgios Kotoulas, Antonios Magoulas, Costas S. Tsigenopoulos, Tereza Manousaki

**Affiliations:** ^1^ School of Medicine, University of Crete, Heraklion, Greece; ^2^ Institute of Marine Biology, Biotechnology and Aquaculture, Hellenic Centre for Marine Research, Heraklion, Greece; ^3^ Department of Ecology, Evolution and Behavior and the National Natural History Collections, The Hebrew University, Jerusalem, Israel

**Keywords:** silver-cheeked toadfish, synteny, phylogenomic analyses, gene families, tetrodotoxin, genome evolution

## Abstract

The Tetraodontidae family encompasses several species which attract scientific interest in terms of their ecology and evolution. The silver-cheeked toadfish (*Lagocephalus sceleratus*) is a well-known “invasive sprinter” that has invaded and spread, in less than a decade, throughout the Eastern and part of the Western Mediterranean Sea from the Red Sea through the Suez Canal. In this study, we built and analysed the first near-chromosome level genome assembly of *L. sceleratus* and explored its evolutionary landscape. Through a phylogenomic analysis, we positioned *L. sceleratus* closer to *T. nigroviridis,* compared to other members of the family, while gene family evolution analysis revealed that genes associated with the immune response have experienced rapid expansion, providing a genetic basis for studying how *L. sceleratus* is able to achieve highly successful colonisation. Moreover, we found that voltage-gated sodium channel (NaV 1.4) mutations previously connected to tetrodotoxin resistance in other pufferfishes are not found in *L. sceleratus*, highlighting the complex evolution of this trait. The high-quality genome assembly built here is expected to set the ground for future studies on the species biology.

## Introduction

The opening of the Suez Canal in 1869 initiated a process of biological invasions from the Red Sea into the Mediterranean, an event commonly known as Lessepsian migration (Por, 1971; Golani and Appelbaum-Golani, 1977). This influx of marine organisms has greatly impacted the local communities in ecological, evolutionary (Sax et al., 2007), and economical terms (Arim et al., 2006). Lessepsian fishes comprise a significant percentage of all recorded invasive species in the Mediterranean Sea (Zenetos et al., 2012) and may be causing several indigenous species displacements (Golani and Appelbaum-Golani, 2010). Lessepsian migration, having both direct and indirect human-driven origins, is a phenomenon that offers a unique opportunity for studying fast evolutionary change (Palumbi, 2001).

One of the most successful Lessepsian migrant species is the silver-cheeked toadfish, *Lagocephalus sceleratus* (Gmelin 1789), a member of the Tetraodontidae family (called puffers), widely distributed throughout the Indian and Pacific Oceans (Akyol et al., 2005). The first record of *L. sceleratus* invasion in the Mediterranean Sea was reported in 2003 in the Gökova Bay, in the south-eastern Aegean Sea coast of Turkey (Filiz and Er, 2004) and 2 years later in the North Cretan Sea (Kasapidis et al., 2007). Since then, is has spread rapidly throughout the entire Levant, Aegean and Ionian Seas (Kalogirou, 2013; Akyol and Ünal, 2017).

The survival and dispersal rate of alien species into novel habitats are dependent on multiple factors including encountering previously unknown pathogens. Thus, the invasion success may be affected by the effectiveness of the immune response (Kolar and Lodge, 2001; Lee and Klasing, 2004) rendering the gene families involved in the adaptive immune response potentially under positive selection.

*Lagocephalus sceleratus* is a highly toxic species, which along with other pufferfishes, contains in different tissues, a toxic organic compound, tetrodotoxin (TTX) (Chua and Chew, 2009; Kosker et al., 2016). TTX is accumulated in high concentration in ovary, liver and intestine of *L. sceleratus* individuals, while lower amounts have been detected in skin and muscle tissues (Akbora et al., 2020). TTX is the metabolic product of TTX-producing bacteria and is retained in the fish tissues. However, the mechanisms of absorption and the differential concentration in the tissues and the organs of pupperfishes remain unclear (Nagashima and Arakawa, 2016). TTX toxicity is due to its binding to the outer pore of the NaV1.4 (SCN4) channel, blocking the transport of sodium ions across the pore (Hanifin, 2010). Multiple pufferfishes have been shown to deploy TTX resistance through mutations in specific domains of NaV1.4, (Venkatesh et al., 2005; Soong and Venkatesh 2006; Jost et al., 2008).

In this study, we provide and analyse the first high-quality genome assembly of *L. sceleratus*. To that end, we combine Illumina and Oxford Nanopore Technology (ONT) reads to construct a highly contiguous assembly, which allows us to explore the genomic landscape of this successful invader and study the genetic background of TTX-resistance in the species. This valuable and robust genome resource will facilitate future studies on the ecology, evolution and potential exploitation of this invasive species.

## Materials and Methods

### Sample Collection and Sequencing

Animal care and handling were carried out following well established guidelines [Guidelines for the treatment of animals in behavioral research and teaching. Anim. Behav. 53, 229–234 (1997)].

One female fish (58 cm in length) was caught alive in Agios Georgios (Hersonissos), Crete, Greece (35°20′07.50″N 25°23′11.30″E) at the pre-spawning/spawning stage (as determined by stereoscopic investigation of the oocytes) and was anesthetized using clove oil. In total, 10 ml of blood was collected using a sterilized syringe and stored in tubes that contained ∼1/10 of volume heparin for subsequent DNA extraction.

DNA extraction for the purpose of ONT sequencing was conducted on the day of sampling, from 2 μl of the freshly collected blood, using Qiagen Genomic tip (20 G) and following ONTs protocol for DNA extraction from chicken blood. The final elution was made with 50 μl AE buffer providing 90.4 ng/μl (Qubit measurement) of high purity, high molecular weight DNA (Nanodrop ratios: 260/280 = 1.87 and 260/230 = 2.12). DNA integrity was assessed by electrophoresis in 0.4% w/v Bio-Rad Megabase agarose gel. We constructed four ligation libraries (SQK-LSK109) following the manufacturer’s instructions. Approximately 1.2 μg of unsheared DNA was used for each library. Two of the prepared libraries were divided into two aliquots. Each library was run for approximately 24 h on a MinION sequencer at HCMR, after which the ONT nuclease flush protocol was performed, and a fresh library or library aliquot was loaded onto the same R9.4.1 flow cell. The total run time was ∼130 h. Basecalling was done with Guppy v3.2.4 (https://community.nanoporetech.com/posts/guppy-3-2-4-release) in High Accuracy Mode with minimum quality score 7.

For Illumina sequencing, we proceeded with 2-days old, refrigerated blood samples using the same DNA extraction protocol as above. We used 4 μl of blood eluted in 100 μl AE buffer which resulted in 79.2 ng/μl (Qubit measurement) of pure DNA (260/280 = 1.85 and 260/230 = 2.21).

Template DNA for Illumina sequencing was sheared by ultrasonication in a Covaris S220 instrument. A PCR-free library was prepared with the Kapa Hyper Prep DNA kit with TruSeq Unique Dual Indexing. Paired end 2 × 150 bp sequencing was performed on an Illumina Hiseq4000 platform.

Tissue samples from brain, gonad, skin, liver, spleen and muscle of the same female individual, were grounded and powdered using pestle and mortar under liquid nitrogen, and except the spleen tissue were all homogenized in TRIzol® reagent (Invitrogen) assisting the homogenization procedure using needle and syringe. Total RNA was extracted from the TRIzol® homogenate according to the manufacturer’s instructions. In the case of spleen, due its nature and the inability to clean properly the RNA from “dirt” even after five washes with ethanol, the spleen RNA was finally extracted using the commercial kit NucleoSpin® RNA. The quantity of the isolated RNA was measured spectrophotometrically with NanoDrop® ND-1000 (Thermo Scientific), while its quality was tested on an agarose gel (electrophoresis in 1.5% w/v). Finally, all samples were used for mRNA paired-end library construction with the Illumina TruSeqTM RNA Sample Preparation Kits v2 following the manufacturer’s protocol (Poly-A mRNA isolation with oligo-dT beads, mRNA fragmentation, followed by transcription into first-strand cDNA using reverse transcriptase and random hexamer primers) and sequenced as 150 bp paired reads in half a lane of a HiSeq4000® following the protocols of Illumina Inc. (San Diego, CA).

### Raw Data Pre-Processing and Genome Size Estimation

Quality assessment of the raw DNA Illumina sequence data was performed with FastQC v0.11.8 (Andrews, 2010). Low quality reads and adapters were removed using Trimmomatic v0.39 (Bolger et al., 2014). The reads were scanned by a 4-based sliding window with an average cutting threshold lower than 15 Phred score. Leading and trailing bases with quality scores less than 10 were also filtered out. Reads with total length shorter than 75 bp and average score below 30 were omitted. The same process was applied to the RNASeq reads.

Adapter trimming and length filtering of basecalled ONT data was done using Porechop v0.2.4 (https://github.com/rrwick/Porechop) with default parameters and the option -- discard_middle to discard reads with internal adapters.

The genome size was estimated using the k-mer histogram method with Kmergenie v1.7051 (Chikhi and Medvedev, 2014) from the Illumina genomic sequencing data.

### *De Novo* Genome Assembly

**FIGURE 1:**
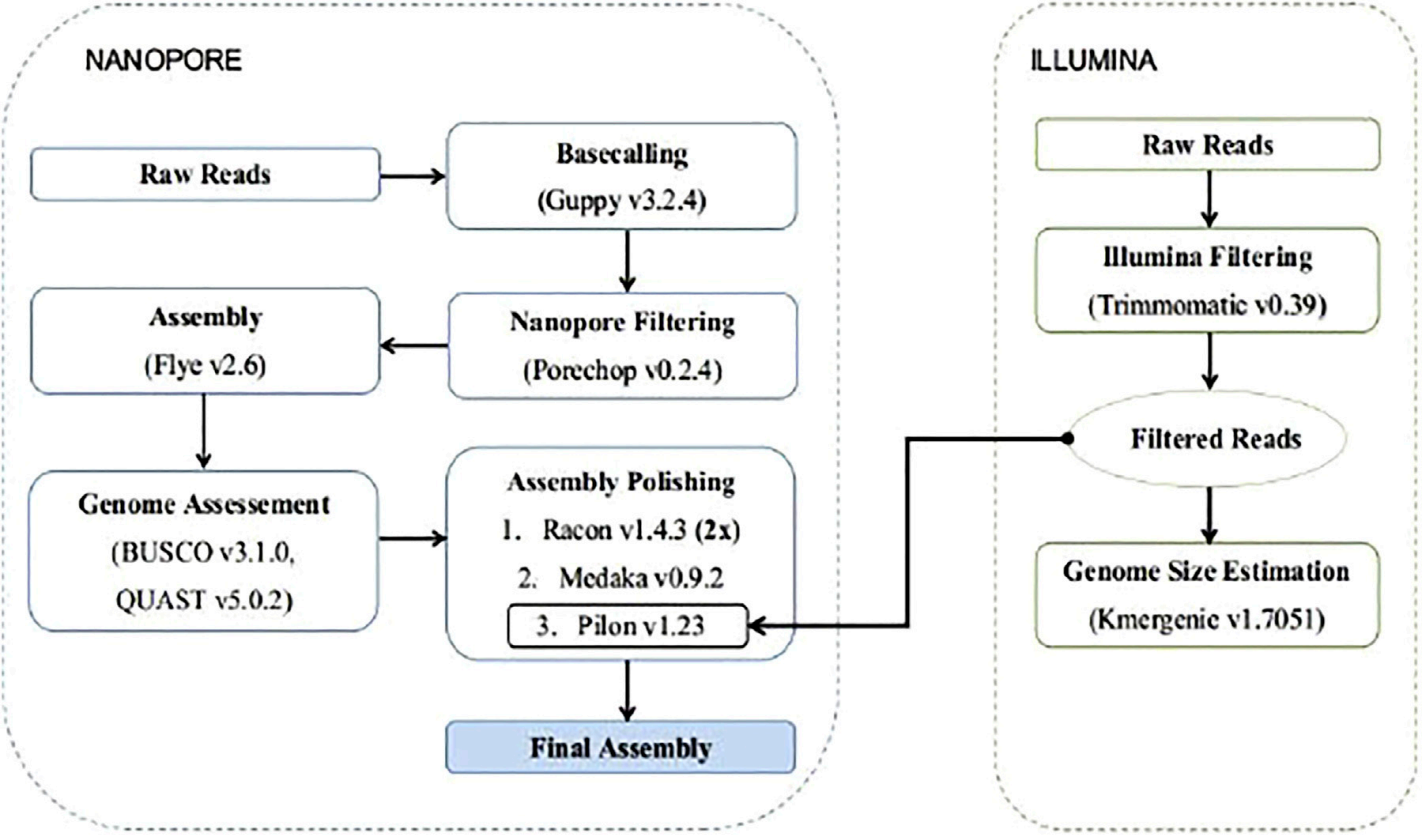
*Lagocephalus sceleratus* genome assembly pipeline.

To build the genome assembly the long ONT reads were used for the construction of an initial *de novo* assembly, and then the Illumina reads were used for the polishing stages. (Figure 1). To construct the initial assembly, we used the v. Flye v2.6 (Kolmogorov et al., 2019) algorithm, a repeat graph assembler. The assembly was evaluated by assessing: 1) the N50 sizes of contigs, using QUAST v5.0.2 (Gurevich et al., 2013), and 2) a gene completeness score using BUSCO v3.1.0 (Simão et al., 2015) against the Actinopterygii ortholog dataset v10, with default parameters. In addition Genomescope2 was used to for kmer frequency profiling (Ranallo-Benavidez et al., 2020).

The produced assembly was polished with two rounds of Racon v1.4.3 (Vaser et al., 2017; Vaser and Šikić, 2020), using the prepossessed long reads mapped against the assembly with Minimap2 v2.17 (Li, 2018). Further polishing was performed with Medaka v0.9.2 (https://github.com/nanoporetech/medaka) and the final polishing was completed using Pilon v1.23 (Walker et al., 2014) after mapping the Illumina reads against the partially polished assembly with Minimap2 v2.17.

### Genome Annotation

#### Repeat Elements Annotation

To identify the repeat elements’ sequences, we constructed *de-novo* repeat library using RepeatModeler2 (Flynn et al., 2020), including RECON v1.08 (Bao and Eddy, 2002), RepeatScout v1.0.6 (Price et al., 2005), LtrHarvest (Ellinghaus et al., 2008), which is incorporated in GenomeTools v1.5.9, Ltr_retriever v2.7 (Ou and Jiang, 2018), assuming default parameters and the extra LTRStruct pipeline which includes Mafft v7.453 (Katoh and Standley, 2013), CD-HIT v4.8.1 (Li and Godzik, 2006) and Ninja v0.95 (Wheeler, 2009). Thereafter, sequences that were obtained by RepeatModeler, were combined with Repbase v17.01 and a custom database constructed with the entries of *Takifugu rubripes*, *Takifugu flavidus,* and *Tetraodon nigroviridis* of the FishTEDB (Shao et al., 2018). Finally, RepeatMasker v4.1.0 (Tarailo-Graovac and Chen, 2009) was used to annotate repeat elements based on the above-described database.

#### Gene Prediction and Functional Annotation

After repeat masking, gene prediction was conducted using MAKER2 pipeline v2.31.10 (Holt and Yandell, 2011) with two iterative rounds. We used a combined strategy of *ab initio*, homology-based and transcriptome-based methods. In the first round, for homology annotation, MAKER2 was initially run in protein2genome mode, while SWISS-PROT (www.uniprot.org) was used for protein sequences extraction of three closely related species, *Mola mola*, *Tetraodon nigroviridis*, and *Takifugu rubripes*. For annotation using the RNA-Seq data, est2genome mode was enabled, which is based on transcriptome evidence. Τranscriptomic reads from all sequenced tissues were mapped and assembled through the genome-guide approach, using HISAT2 v2.2.0 (Kim et al., 2015) and StringTie v2.1.1 (Pertea et al., 2015). *Ab initio* prediction was performed with SNAP (Korf, 2004) (http://korflab.ucdavis.edu), which was independently trained on *L. sceleratus* genome with default parameters and AUGUSTUS v3.3.3 (Stanke et al., 2006) previously trained through BUSCO v3.1.0 (Simão et al., 2015) with the extra parameter “-long.” The second round of MAKER2 was run using the previously trained models with the same settings as round one, except est2genome and protein2genome modes. The previous custom repeat library and MAKER2 repeat library that used for genome masking, remained for both rounds. The completeness of putative genes was assessed using BUSCO v4.0.5 (Simão et al., 2015) against the Actinopterygii odb10 database.

The functional annotation of the predicted genes of *L. sceleratus* was performed by similarity search against the UniprotKB/Swissprot database (release-2020_03) with BLASTP v.2.9.0+ (e-value 1e-6, -max_target_seqs = 10) (Altschul et al., 1990). InterProScan v5 (Jones et al., 2014) was used to search motifs and domains against all default databases and the extra of SignalP_EUK and TMHMM. Functional annotation results were also retrieved using eggNOG-mapper (Huerta-Cepas et al., 2017) based on fast orthology assignments using precomputed eggNOG v5.0 (Huerta-Cepas et al., 2019) clusters and phylogenies.

#### Gene Ontology Mapping

Gene ontology analysis was carried out using a custom python script (gene_ontology_mapping.py). Gene ontology terms were retrieved through the Uniprot API service (https://www.uniprot.org/help/programmatic_access) and as queries we chose the best blast hits that we extracted after the functional annotation step against UniProtKB/Swiss-Prot.

### Phylogenomic Analysis

#### Orthology Assignment

To identify paralogous and orthologous genes, we compared 30 whole-genome protein-coding gene sets from teleost fish (Supplementary Table S3) (27 species from our previous dataset of Natsidis et al. (2019) in addition with two extra Tetraodontidae species i.e., *Takifugu bimaculatus. T. flavidus*, and *L. sceleratus*) with Orthofinder v2.3.12 (Emms and Kelly, 2015) using default parameters. Firstly, the longest isoform of each gene was kept using the *primary_transcript.py* script provided by Orthofinder suite. For *L. sceleratus,* only the longest isoforms over 30 amino acids were extracted with a custom script (longestIsoforms.py) and used in the analysis.

#### Species Tree Reconstruction

For the phylogenomic analysis the orthogroups produced by orthofinder were filtered, keeping those containing a single gene per species to avoid inclusion of paralogs. Then, we kept those with representation from at least 26 out of the 30 taxa analysed in total, using a custom python script (filtered_orthogroups.py) The amino-acid sequences encoded by selected genes of each orthogroup were aligned using MAFFT v7.453 (Katoh and Standley, 2013), with the -auto mode. The aligned orthogroups were then concatenated using a python script by P. Natsidis (https://github.com/pnatsi/Sparidae_2019/blob/master/concatenate.py). The resulted alignments were filtered with Gblocks v0.91b (Castresana, 2000) to exclude poorly aligned regions with the following parameters: “Allowed Gap Positions” was set to half, “Minimum Length of a Block” was set to 8, “Minimum Number of Sequences for a Flanking Position” was set to 20, and “Minimum Number of Sequences for a Conserved Position” was set to 18.

Then, we ran RAxML-NG v0.9.0 (Kozlov et al., 2019) for phylogenetic tree reconstruction, and in order to select the best model, we used ModelTest-NG v0.1.6 (Darriba et al., 2020) specifying the --topology type parameter to maximum likelihood (ml) mode. The phylogenomic inference was run using the selected model, JTT + I + G4 + F. To assess the branch confidence, we ran 100 bootstrap replicates. The final tree was visualized using R/RStudio RStudio Team (2021) with a custom script using *Lepisosteus oculatus* as outgroup (phylo_tree_plot.r).

### Gene Family Expansion and Contraction

The expansion and contraction of gene families were analysed using CAFE v4.2.1 (De Bie et al., 2006). The sequences were first clustered using MCL (Enright et al., 2002) following the CAFE developers’ instructions (https://iu.app.box.com/v/cafetutorial-files) and filtering the gene families using a custom python script (cafe_filterin_blast_dump.py) to exclude gene families that contain at least one or more species with ≥100 genes. An ultrametric tree was produced with r8s v1.81 (Sanderson, 2003) using the phylogenetic tree produced in our phylogenomic analysis and the divergence time for *Thunnus thynnus* and *Oreochromis niloticus* taken from TIMETREE (http://www.timetree.org/). Finally, CAFE was run with conditional *p*-values, for each gene family below 0.01.

### Synteny Analysis

Synteny analyses were performed on two tiers, on whole-genome sequence comparison and at the gene level.

For the whole-genome comparison, the *L. sceleratus* genome assembly was compared with all available genomes of Tetraodontiformes to date (*T. nigroviridis*, *T. rubripes*, *T. flavidus* and *T. bimaculatus*). Furthermore, we performed similar comparisons aligning the genomes of *T. nigroviridis and T. rubripes* against the other species. For the alignment, we used LAST v1145 (Kielbasa et al., 2011), implementing the sensitive alignment protocol as described for Human-mouse whole-genome project comparison (https://github.com/mcfrith/last-genome-alignments) with e-value cut-off 0.001. On the gene level, we used the one-to-one orthologues outputs of Orthofinder v2.3.12 analysis for comparing the same set of species.

We selected for visualization the 41 largest contigs of the *L. sceleratus* assembly representing ∼91% of the genome (Supplementary Figure S1), for both whole-genome and gene-based synteny results. In each set of fish, the whole-genome pairwise alignments were plotted by custom python scripts (synteny_plot.py) (Supplementary Figures S3–S8), while the one-to-one orthologs relationships of all the above-mentioned comparisons, were visualized through Circos (circos_plot.py) (Krzywinski et al., 2009) (Supplementary Figures S9–S13).

### Alignment of Voltage-Gated Sodium Channel Alpha Subunit 4 (*SCN4A*)

Mutations in SCN4A proteins have been previously associated with pufferfish TTX resistance (Venkatesh et al., 2005). To investigate these mutations further, sequences of the SCN4 alpha subunit were identified *via* BLAST (reciprocal best hit approach) against *D. rerio*, *L. sceleratus* and other fishes from the species tree (Supplementary Table S3), as well as *Takifugu pardalis* (Genbank: BAA90398.1), *Latimeria chalumnae* (NCBI Reference: XP_006003324.1), *Callorhinchus milii* (NCBI Reference: XP_007907351.1), using the *D. rerio* SCN4AA and SCN4AB sequences as queries. The retrieved protein sequences were aligned using MAFFT v7.453 (Katoh and Standley, 2013) with the -auto mode. The alignment was manually curated using Jalview v2.11.1.4 (Waterhouse et al., 2005).

## Results

### Genome Size and Assembly Completeness

Sequencing yielded 57.30 Gb of raw Illumina reads and 9.68 Gb of ONT reads above Q7, with N50 of 48.85 Kb. The estimated genome size was calculated *via* kmergenie (Supplementary Figure S1) and found to be ∼360 Mb and the best predicted k = 81. After quality trimming and filtering, we retained 44.45 Gb of Ιllumina data for genome polishing and 9.67 Gb of ONT data (Table 1) for the genome assembly. The final assembled and polished genome contained 235 contigs with total length of ∼373 Mb, with 41 contigs representing ∼91% of the genome and the largest contig sizing 17 Mb and N50 of 11 Mb (Table 2). In addition, kmer frequency profiling showed low levels of heterozygocity (0.521983%), as calculated using Genomescope2 (Supplementary Figure S2). Regarding genome completeness, we found 98% (4,513 out of 4,584) of the genes included in the BUSCO Αctinopterygian gene set. Of those, 96.20% (4,410) were complete (Table 2), suggesting a high level of completeness and contiguity in the built assembly.

**TABLE 1:** Summary of sequencing results.

Sequencing technology	Raw reads	Quality-controlled reads	Coverage
Illumina	57,303,140	44,475,382	38 x
MinION	552,476	484,152	20 x

**TABLE 2:** Polished genome assembly statistics and completeness.

Total contigs	235
Total contig sequence	373,851,781 bp
GC (%)	46.7
Contig N50	11,297,640 bp
Contig N75	6,386,954 bp
Longest contig	17,085,954 bp
Contig L50	14
Contig L75	25
BUSCO completeness score	
Complete	96.60%
Single	95.70%
Duplicated	0.90%
Fragmented	1.00%
Missing	2.40%
Total number of Actinopterygii orthologs	3,640 (97.60%)

### Repeat Annotation, Gene Prediction and Functional Annotation

A total of 61.9 Mb of repeat sequences that accounted for 16.55% of the genome assembly were masked in *L. sceleratus*. The class of Retroelements makes up 7.81% of the total assembly and LINEs are the most abundant of this class, with 5.54%. LTR elements sequences (2.07%) is the second most abundant group in the Retroelements class, and the results also indicated that 2.30% of the genome assembly consists of sequences of the class DNA transposons (Table 3).

**TABLE 3:** Repeat elements annotation statistics.

Repetitive elements	Number of elements	Length occupied (bp)	Percentage of sequence (%)
Retroelements	204,205	29,198,482	7.81
SINEs	5,447	743,745	0.20
Penelope	3,627	2,572,446	0.69
LINEs	171,108	20,706,770	5.54
CRE/SLACS	0	0	0.00
L2/CR1/Rex	45,521	7,208,493	1.93
R1/LOA/Jockey	573	179,409	0.05
R2/R4/NeSL	48,675	3,708,881	0.99
RTE/Bov-B	47,510	3,856,263	1.03
L1/CIN4	16,080	2,144,919	0.57
LTR elements	27,650	7,747,967	2.07
BEL/Pao	410	259,661	0.07
Ty1/Copia	235	96,868	0.03
Gypsy/DIRS1	13,595	3,917,10	1.05
Retroviral	4,582	1,298,868	0.35
DNA transposons	59,679	8,587,997	2.30
hobo-Activator	25,182	2,911,788	0.78
Tc1-IS630-Pogo	14,227	2,885,165	0.77
En-Spm	0	0	0.00
MuDR-IS905	0	0	0.00
PiggyBac	644	131,052	0.04
Tourist/Harbinger	2,399	392,537	0.10
Other (Mirage, P-element, Transib)	99	5,804	0.00
Unclassified	113,777	1,126,996	5.97
Small RNA	0	0	0.00
Satellites	20	85,955	0.02
Simple repeats	9,305	1,126,996	0.30

### Gene Prediction and Functional Annotation

The combination of *ab initio*-based, homologue-based and RNASeq-based methods resulted in 32,451 putative protein-coding genes. After removing putative genes with Annotation Edit Distance (AED) (Eilbeck et al., 2009) score below one (AED<1), with custom script (longestIsoforms.py), we ended up with 21,251 genes of average gene length and exon size 587,43 and 249,62 bp, respectively. A total of 20,578 genes were successfully annotated, accounting for 97% of the predicted gene set (Table 4). Using BLAST to search the longest proteins encoded by each locus, a total of 17,706 *L. sceleratus* genes had a match in *T. rubripes*, and 16,706 in *T. nigroviridis.* Of those matches, we found 14,271 reciprocal best hits in *T. rubripes* and 13,425 reciprocal best hits in *T. nigroviridis*.

**TABLE 4:** Summary statistics of functional annotated protein-coding genes.

Type	Number	Percent (%)
Blast	18,805	88
InterProScan	20,347	96
EggNog-Mapper	17,849	84
Predicted genes	20,578	97
Total Genes	21,251	

The completeness of the gene set was assessed using BUSCO v4.0.5 (Simão et al., 2015). From a core set of 3,640 single-copy ortholog genes from the *Actinopterygii* (odb 10) lineage, 92.2% were complete (70.5% as single-copy, 21.7% as duplicates), 1.7% were fragmented and 6.1% were not found.

### Orthology Assignment and Phylogenomic Analysis

The total number of genes from all 30 fish proteomes (Supplementary Table S3) analysed by Orthofinder was 731,383 while 21,897 orthogroups were identified. After filtering, we chose 731 one-to-one orthogroups to construct the super-alignment. The initial matrix consisted of 494,732 amino acid positions. The Gblocks-filtered matrix contained 252,477 positions (51% of the initial), which were used for the phylogenomic analysis.

**FIGURE 2:**
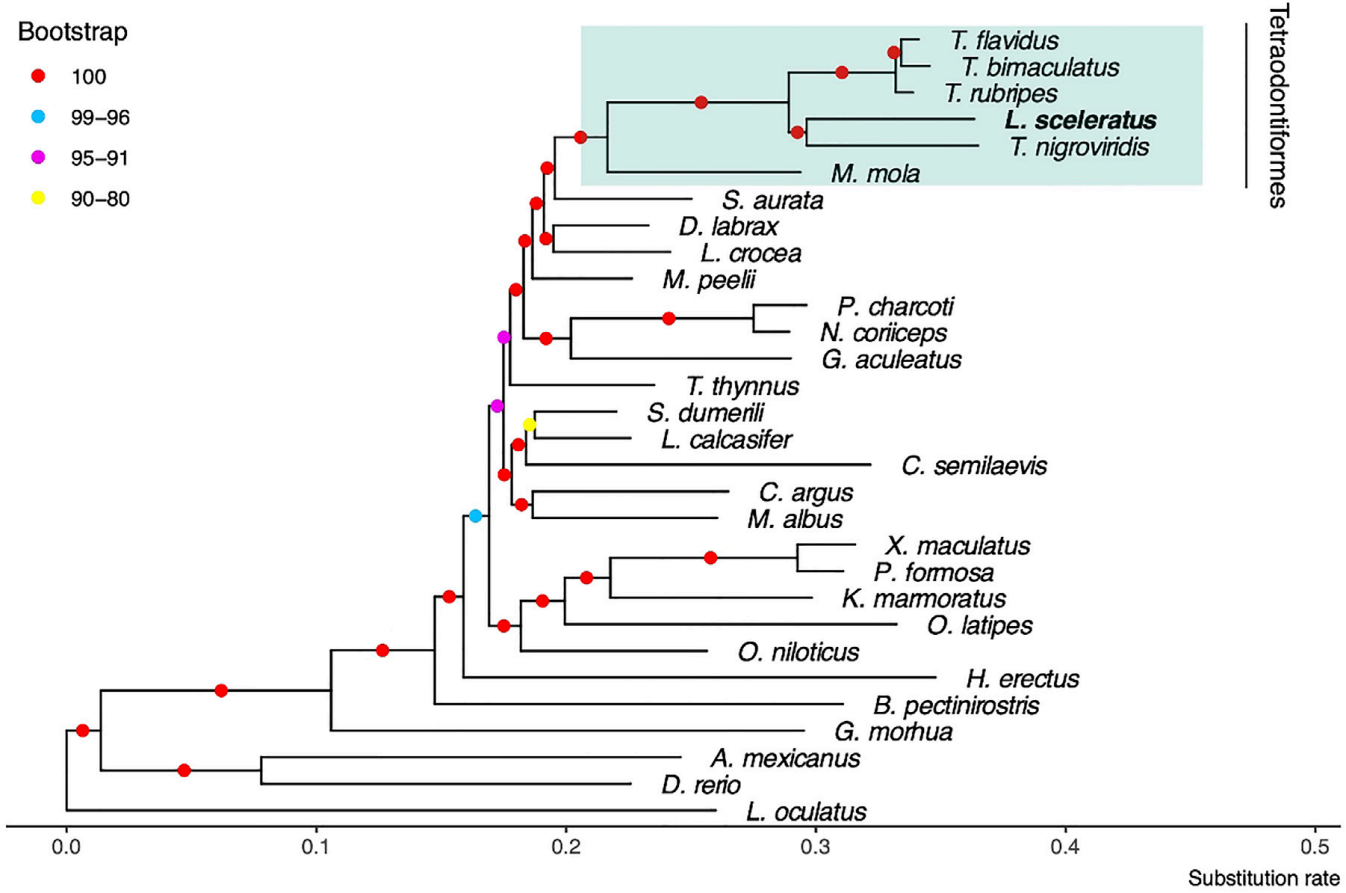
Maximum likelihood tree using JTT + I + G4 + F model and 100 bootstrap replicates. The spotted gar *L. oculatus* was used as outgroup.

We identified JTT + I + G4 + F as the best model which was used for the estimation of the phylogenetic tree (Figure 2). At the resulted phylogeny, almost all branches were supported with 100 bootstraps. The recovered phylogenetic position of *L. sceleratus* is within Tetraodontidae clade and is placed closer to *T. nigroviridis.*

### Gene Family Evolution

**FIGURE 3:**
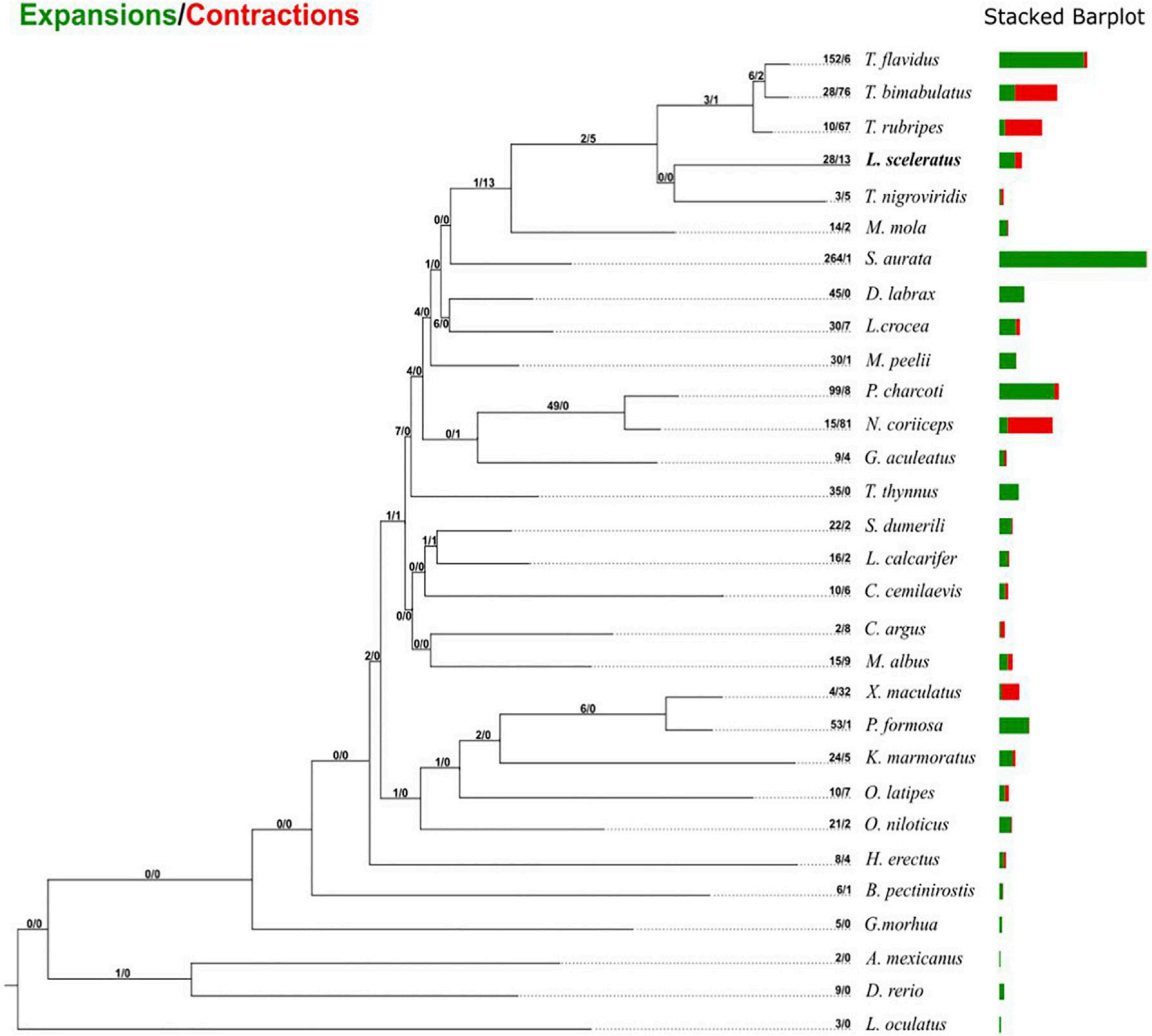
Gene family gain-and-loss analysis, including the number of gained gene families (green) and lost gene families (red). The stacked barplot on the right indicates the gains and losses per species.

Gene family evolutionary analysis revealed multiple predicted rapidly expanded and contracted gene families of *L. sceleratus* (Figure 3; Supplementary Tables S4, S5), respectively. Gene families found only in one or more pufferfish species and not in any other taxon were considered as puffer-specific. Among rapidly evolving families, four gene families were identified as *L. sceleratus* specific, two as rapidly expanding (Supplementary Table S1) and two gene families as rapidly contracting. Finally, 43 *T. bimaculatus* and 45 *T. rubripes* families were found to be rapidly contracted (Supplementary Figures S1–S2). The gene family analysis did not reveal a core set of rapidly evolving families at the base of all five puffer species (Supplementary Figures S2–S3).

### Synteny Analysis

#### Whole Genome-Based Synteny

**FIGURE 4:**
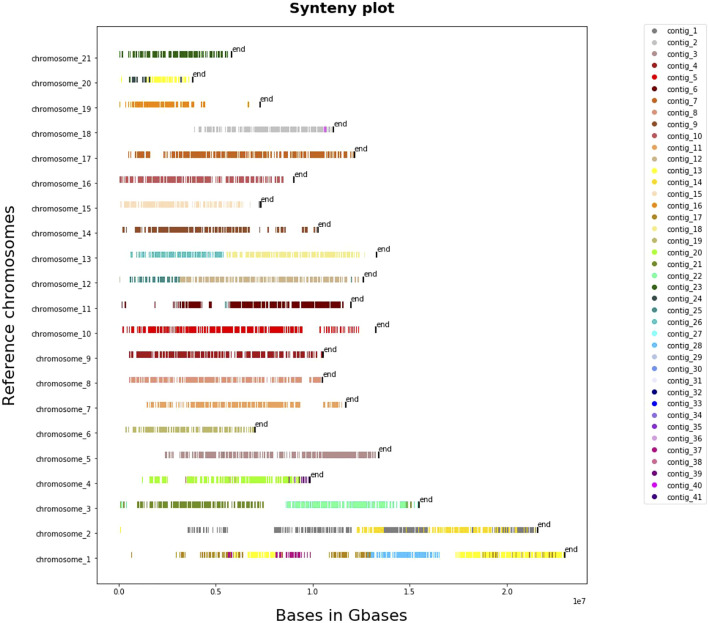
Synteny plot of pairwise whole genome alignment of *L. sceleratus* against *T. nigroviridis.* The plot illustrates the contigs of the *L. sceleratus* aligned to *T. nigroviridis* chromosomes (y-axis), grouped by a specific color which are represented on the legend right to the plot.

The results that we obtained from whole-genome pairwise alignment of *L. sceleratus* against the other four puffers (*T. nigroviridis, T. rubripes, T. flavidus and T. bimaculatus*) are summarised in Supplementary Table S2. Comparisons of *L. sceleratus* against *T. nigroviridis* (Figure 4), *T. rubripes, T. flavidus* and *T. bimaculatus* (Supplementary Figures S9–S11) indicated high collinearity of the studied species’ genome against the rest. In particular, we aligned the 41 largest contigs of *L. sceleratus* against the chromosomes of *T. flavidus* (∼231.3 aligned Mb), *T. bimaculatus* (∼224 aligned Mb), *T. rubripes* (∼227 aligned Mb) and *T. nigroviridis* (∼154.7 aligned Mb).

Whole genome alignments were also carried out between *T. nigroviridis* and *T. flavidus*, as well as *T. bimaculatus* and *T. rubripes* (Supplementary Figures S4–S6) revealing highly contiguous matches across all species, with higher collinearity observed between *T. nigroviridis* and *T. bimaculatus* compared to *T. nigroviridis* and *T. rubripes*.

#### Gene-Based Synteny Analysis

**FIGURE 5:**
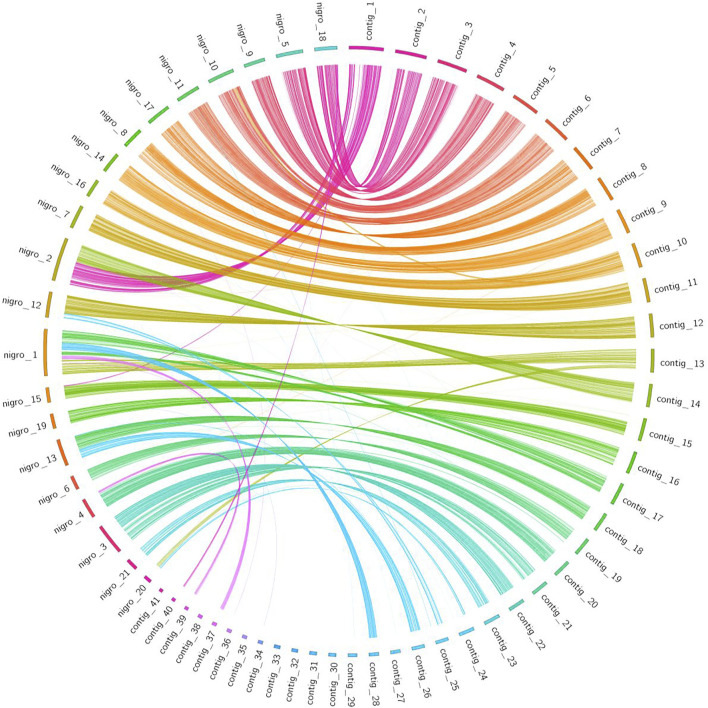
Circos plot illustrating syntenic relationships between *L. sceleratus* contigs (right) against *T. nigroviridis* chromosomes (left), based on one-to-one orthologous genes. Ribbons link orthologous genes between the two species and colors represent the different contigs of *L. sceleratus*.

**FIGURE 6:**
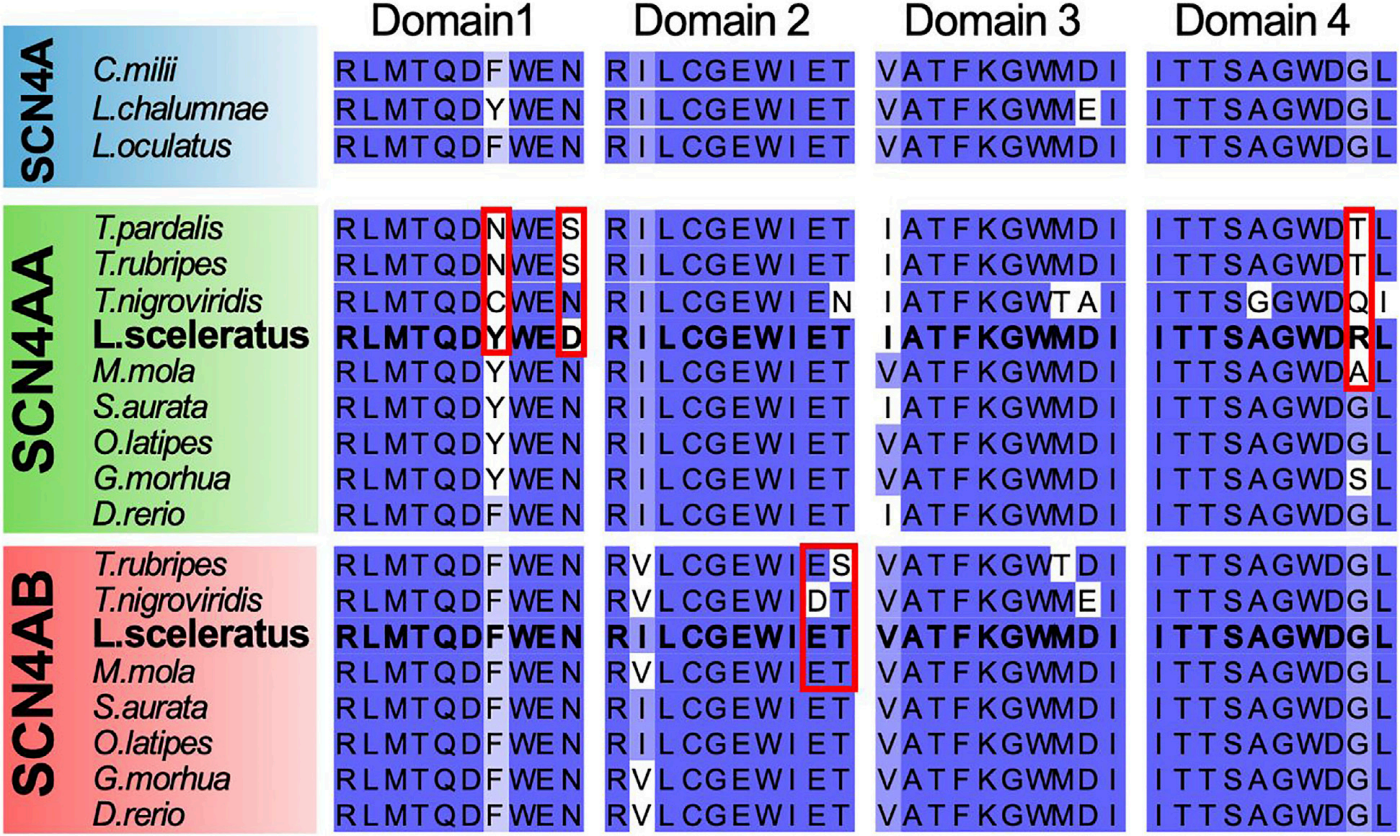
Multiple alignment plot of the four Ion Transport domain sequences previously studied by Venkatesh et al. (2005), from *Latimeria chalumnae* (NaV1.4), *Lepisosteus oculatus* (NaV1.4), *Callorhinchus milii* (NaV1.4), *T. pardalis* (NaV1.4a), *T. rubripes* (NaV1.4a, NaV1.4b), *T. nigroviridis* (NaV1.4a, NaV1.4b), *L. sceleratus* (NaV1.4a, NaV1.4b), *M. mola* (NaV1.4a, NaV1.4b), *S. aurata* (NaV1.4a, NaV1.4b), *O. latipes* (NaV1.4a, NaV1.4b), *M. morhua* (NaV1.4a, NaV1.4b), and *D. rerio* (NaV1.4a, NaV1.4b). Residues outlined in red, have been previously associated with TTX resistance in *T. rubripes and T. nigroviridis* by Venkatesh et al. (2005).

Synteny information obtained from a gene-based analysis revealed higher conserved synteny between *L. sceleratus* and *T. nigroviridis* (Figure 5) than between *L. sceleratus* and *T. rubripes* or *T. bimaculatus* (Supplementary Figures S14–S15). *L. sceleratus*’ contigs that are unaligned to *T. nigroviridis* chromosomes in Figure 6, were aligned to unplaced regions of the *T. nigrorviridis* assembly (Supplementary Figure S6).

### *L. sceleratus* Lacks SCN4A Mutations Previously Associated With Pufferfish TTX Resistance

The SCN4A protein has been previously associated with pufferfish TTX resistance, showing that mutations in the core of its ion transport domain led to changes in TTX binding affinity. We performed multiple sequence alignment of the ion transport domain sequences from different Tetraodontiformes species and selected outgroups, including teleosts (SCN4AA and SCN4AB paralogs) and other vertebrates. We found that *L. sceleratus* and *M. mola* lack the previously characterized mutations in domains 1 and 2 in residues outlined in red. In contrast, all Tetraodontiformes show changes in the second to last residue of the domain 4 sequence outlined in red in Figure 6.

## Discussion

### Genome Size and Assembly Completeness

In this study, a high-quality pufferfish genome assembly of high contiguity was reconstructed, from data obtained from a single MinION flow cell and half a lane of Illumina HiSeq. To our knowledge, only one other highly contiguous reference Tetraodontiformes genome assembly has previously been constructed using the same strategy, for *Thamnaconus septentrionalis* (Bian et al., 2019). The assembly of *L. sceleratus* (360 Mb) is consistent with the predicted genome size from kmergenie and is comparable in size with that of other puffers, such as *Fugu rubripes* (∼365 Mb; Aparicio et al., 2002), *T. flavidus* (∼377 Mb; Zhou Y. et al., 2019), *T. bimaculatus* (∼393.15 Mb; Zhou Z. et al., 2019), *T. obscurus* (∼373 Mb; Kang et al., 2020) and *T. nigroviridis* (340 Mb, Jaillon et al., 2004). The contig N50 value (∼11 Mb) of the *L. sceleratus* assembly is considerably greater than that reported for the genomes of *T. bimaculatus* (1.31 Mb; Zhou Z. et al., 2019) and *T. flavidus* (4.4 Mb; Zhou Y. et al., 2019). Similarly, our assembly appears of equivalent levels of completeness to other Tetraodontidae genomes, based on BUSCO scores (e.g., *T. obscurus* (Kang et al., 2020) and *T. flavidus* (Zhou et al., 2019a)].

### Repeat Content, Gene Prediction and Functional Annotation

The percentage of transposable elements (TEs) found in the *L. sceleratus* genome (16.55% of the assembled genome) is marginally higher than the one found in *T. septentrionalis* (14.2%) (Bian et al., 2019), *T. obscurus* (11.05%) (Kang et al., 2020), and *M. mola* (11%) (Pan et al., 2016). Moreover, it is almost twofold higher that in *T. rubripes* (7.53%) and threefold higher than *T. nigroviridis* (5.60%) and *T. flavidus* (6.87%) (Gao et al., 2014). *T. rubripes* contain more copies of transposable elements than *T. nigroviridis,* which have been proposed to contribute to its marginally larger genome size (365–370 Mb) (Jatllon et al., 2004). Although the *L. sceleratus* genome has a comparable size to reported *Takifugu* genomes, it harbors much higher repeat content. Moreover, *D. holocanthus* genome of the Diodontidae family contains 36.35% repetitive sequences, almost double the repeat content of *L. sceleratus*. These findings imply that TEs might follow an independent pathway of accumulation and diversification across Tetraodontiformes species. In the case of *L. sceleratus*, such differential repeat expansion may have taken place after the divergence of the Takifugu and Tetraodon genera.

Despite such TE content variation across closely related taxa, positive correlation of genome size and TE repeat content has been documented across a larger evolutionary scale in teleosts (Shao et al., 2019). For example, the relatively smaller genome of *T. nigroviridis* (∼360 Mb) contains 5.6% TEs, in contrast to the zebrafish genome (∼1.4 Gb) which is composed of 55% repetitive sequences (Shao et al., 2019). This positive correlation is also reflected in the small size and relatively low repeat content of the *L. sceleratus* genome, regardless of differences with other pufferfish. However, it would be interesting to further explore these differences, as they may be informative for genome evolution. As an interesting example, LINE elements are the most abundant in the *L. sceleratus* genome, with ∼170,000 copies, as compared to the ∼12,300 copies of the *T. rubripes* genome. This finding indicates dynamic genome evolution in the two species. Previous studies have shown a correlation between genome TEs and species adaptations to new environments, suggesting they may be associated to invasiveness (Stapley et al., 2015; Yuan et al., 2018). Thus, the repeat content of *L. sceleratus* may play a role in its fast adaptation to novel environments and should be investigated further.

### Species Tree Reconstruction

Although the order Tetraodontiformes is a cosmopolitan taxonomic group that includes multiple families, large parts of their phylogenetic relationships remain unexplored. In this study, we presented the first phylogenetic tree based on whole genome data including the invasive “sprinter*” L. sceleratus*. The recovered phylogenetic position of *L. sceleratus* is within Tetraodontidae and is placed closer to *T. nigroviridis*, while the long branch length of the Tetraodontidae clade possibly suggests a faster evolutionary rate. Regarding relationships within the pufferfish group (*T. nigroviridis, T. rubripes, T. flavidus, T. bimaculatus* and *L. sceleratus*), the resulting topology agrees with previous studies (Yamanoue et al., 2011; Meynard et al., 2012; Hughes et al., 2018; Hughes et al., 2021). Moreover, the Tetraodontidae group was recovered confidently as monophyletic in accordance with Yamanoue et al. (2011). Our results suggest that Tetraodontiformes are the closest group to Sparidae and corroborates the results of Natsidis et al. (2019) and of others (Kawahara et al., 2008; Meynard et al., 2012), based both on six mitochondrial and two nuclear genes.

### Synteny Analysis

All pairwise comparisons of the whole-genome alignment analysis of *L. sceleratus* against the four other Tetraodontidae species (Figure 5) (Supplementary Figures S9–S11), showed highly conserved synteny. The genome that exhibited the highest synteny conservation with the *L. sceleratus* genome was that of *T. nigroviridis,* in accordance with our reconstructed phylogeny which places the two species as more closely related to each other compared to the rest.

The synteny between *L. sceleratus* and the three species of the genus *Takifugu* (*T. rubripes*, *T. bimaculatus* and *T. flavidus*) was less conserved, especially between *L. sceleratus* and *T. bimaculatus*.

To sum up, the higher synteny between *L. sceleratus* and *T. nigroviridis* corroborates their closer phylogenetic position compared to the three *Takifugu* species.

### Gene Family Evolution and Adaptation

Adapting to a new habitat is a challenging task for a species, requiring a certain degree of physiological plasticity. To achieve establishment in a new niche, an invader must face environmental challenges that involve both biotic and abiotic factors (Crowl et al., 2008). Invasive species are facing novel pathogens during the colonisation of new environments and the ability to deal with these new immune challenges is key to their invasive success (Lee and Klasing, 2004). Interestingly, we found several expanded immune related families, including *immunoglobulins* (*C-Type* and *V-Type*), *Ig heavy chain Mem5-like*, *B-cell receptors* and the *Fish-specific NACHT associated domain,* which are related to the innate immunity (Stein et al., 2007).

In addition, we also detected major histocompatibility complex (*MHC*) *class I* genes in the expanded gene families. MHC genes are crucial for the immune response, involved in pathogen recognition by T cells (Germain, 1994), thus initiating the adaptive immune response. The expanded repertoire of *L. sceleratus* immune response associated genes might be related to its survival in novel habitats, through the detection and inhibition of a wide range pathogens. Therefore, in this context, we suggest further research to explore the role of the expanded genes related to immune response.

Another interesting finding was the expansion of the fucosyltransferase (FUT) gene family. In particular, we detected 24 FUT9 [alpha (1.3) fucosyltransferase 9] genes. Glycosylation is one of the most frequent post-translational modifications of a protein. Many proteins involved in the immune response are glycosylated, extending their diversity and functionality (Bednarska et al., 2017). Fucosylation, a type of glycosylation, plays an essential role in cell proliferation, metastasis and immune escape (Jia et al., 2018). In mice, FucTC has been shown to regulate leukocyte trafficking between blood and the lymphatic system, after its engagement in selectin ligand biosynthesis (Maly et al., 1996).

Overall, based on our results, we believe that the expanded innate immune system gene families identified herein deserve further study as to whether they have contributed to the ability of *L. sceleratus* to spread rapidly (Kalogirou, 2013).

### TTX Resistance of *SCNA4* Sodium Channel

Sodium channels (NaV) are formed by an *α* subunit consisting of four domains (I-IV) and an optional *β* subunit that may alter its activity (Yu et al., 2005). Furthermore, the third whole-genome duplication of teleosts has expanded the *α* subunit family to eight members (Zakon et al., 2011). NaV channels are the target of several neurotoxins (Daly, 1995; Fry et al., 2009) and TTX is known to bind to the outer pore of the NaV1.4 (SCN4) channel, blocking the transport of sodium ions across the pore (Hanifin, 2010). As different pufferfishes have been shown to acquire TTX resistance through mutations in specific domains of NaV1.4 (Venkatesh et al., 2005; Soong and Venkatesh, 2006; Jost et al., 2008), we checked if this is also the case for *L. sceleratus*. For this purpose, we investigated whether the sequences of voltage-gated sodium channels (SCN4AA, SCN4AB) in *L. sceleratus* carry the previously reported mutations associated with TTX resistance in other pufferfish. According to these studies (Venkatesh et al., 2005; Soong and Venkatesh, 2006), the residues that are associated with TTX resistance are located in the same position in NaV1.4a Domain I and were mutated to *Cys* and *Asn*, in *T. nigroviridis* and *T. rubripes*, respectively (Figure 6). Surprisingly, despite the fact that the Domain I mutations lead to extensive decrease in TTX binding (Satin et al., 1992; Kaneko et al., 1997; Yotsu-Yamashita et al., 2000; Venkatesh et al., 2005) and have been associated with TTX resistance in pufferfishes and in one *C. pyrrhogaster* newt (Kaneko et al., 1997), we did not observe any of these reported mutations in the SCN4A_A gene of *L. sceleratus* (Figure 6). Similarly, no changes from the ancestral sequence of the same paralog were found in *M. mola*, which is also a toxin resistant species (Halstead, 1988). The absence of mutations in *L. sceleratus* and *M. mola*, in conjunction with the updated Tetraodontiformes relationships presented in Figure 3, suggest that the changes seen in *T. rubripes* and *T. nigroviridis* have arisen *via* convergent evolution, a hypothesis further supported by the different amino acid replacement observed in these two species. This could also suggest that the position of the replacement is more important than the amino acid change *per se*. Our results are also congruent with previous findings for *Hapalochlaena lunulata* (Geffeney et al., 2019) and *Hapalochlaena maculosa* (Whitelaw et al., 2020), as NaV1.4a Domain I is highly conserved in both octopod species. Furthermore, unlike the pufferfishes *A. nigropunctatus* and *T. nigroviridis* (Jost et al., 2008), the garter snake *Thamnophis couchii* (Feldman et al., 2012) and the octopuses *Hapalochlaena lunulata* (Geffeney et al., 2019) and *Hapalochlaena maculosa* (Whitelaw et al., 2020), replacements were also not observed in the NaV1.4a Domain III of *L. sceleratus*. Similar to *L. sceleratus,* mutated residues are not found in *T. rubripes* either. However, in NaV1.4a Domain IV all the Tetraodontiformes species have a replacement in the same position (outlined in red in Figure 7). Interestingly, a different mutation is observed in each species, except for *T. rubripes* and *T. pardalis,* which both share a change to *Thr*. Similar to what is discussed earlier for the changes observed in domain I, the position of the replacement may be more crucial than the specific changes observed. This could hint to a decrease in TTX binding by disturbing the conserved ancestral binding interface, as previously shown in domain I mutations in *T. rubripes* and *T. nigrovidis* (Venkatesh et al., 2005; Soong and Venkatesh, 2006)*.* Previously, these mutations for *T. rubripes* and *T. nigroviridis* had not been associated with TTX resistance (Venkatesh et al., 2005; Soong and Venkatesh, 2006). At the last positions of Domain IV, there are also two replacements to *His* and *Ser* in *H. lunulata* (Geffeney et al., 2019) and *H. maculosa* (Whitelaw et al., 2020), which may inhibit TTX binding.

The same studies (Venkatesh et al., 2005; Soong and Venkatesh, 2006; Jost et al., 2008) also showed that an *Asp* mutation in Domain II of *T. nigroviridis* NaV1.4b is highly associated with TTX resistance. A similar substitution has been reported in the soft-shelled toxin-resistant clam *M. arenaria* (Bricelj et al., 2005). Strikingly, the *L. sceleratus* and *M. mola* NaV1.4b sequences also lack mutations in Domain II, comparable to NaV1.4a domain I (Figure 6).

While the origin of TTX resistance remains elusive, several studies have provided insight into resistance mechanisms. Toxin tolerance may appear through mutations in sodium channels or toxin binding proteins (Ho et al., 1994; Zou, 2020). A wide range of different organisms have taken advantage of mutations which confer TTX tolerance either independently or complementary. The lack of previously characterised pufferfish mutations associated with TTX resistance in *L. sceleratus* raises several questions about how TTX resistance has evolved. Nevertheless, the results of our analysis imply that combined effects of complex polygenic adaptations working redundantly have played a role in the evolution of this complex trait, while similar genetic changes have arisen convergently multiple times.

## Conclusion

Invader fishes, such as *L. sceleratus,* often thrive in novel environments. Our analysis provides the first high-quality genome assembly built from sequencing a single female individual and a comprehensive evolutionary genomic analysis of the species. We uncovered a close phylogenetic position of *L. sceleratus* with *T. nigroviridis,* untangling relationships within the pufferfish group, that were not clearly resolved in previous studies. The study gives insights into a variety of genomic signatures that may be associated with *L. sceleratus* invasion and colonisation effectiveness. Surprisingly, examination of voltage-gated sodium channels (NaV1.4) revealed a lack of TTX resistance associated mutations found in other pufferfishes, highlighting the complex evolution of the trait. Overall, the *L. sceleratus* genome will be an invaluable resource for additional studies on immune response in novel environments, osmoregulation, reconstruction of ancient chromosome rearrangements, investigation of complex TTX resistance mechanisms and population genomics and adaptation. Such studies are expected to elucidate the mechanisms behind the high invasiveness of *L. sceleratus* and assist the management of this invasive sprinter in the Mediterranean.

## Data Availability

Genomic and transcriptomic Illumina and Oxford Nanopore raw data can be accessed in ENA with the IDs ERX6020380, ERS6677531 and ERR6391424 respectively. The genome assembly has been uploaded with the ENA ID CAJVRN010000000.

## References

[B1] Akbora H. D. Kunter İ. Erçeti̇n T. Elagöz A. M. Çi̇çek B. A. (2020). Determination of Tetrodotoxin (TTX) Levels in Various Tissues of the Silver Cheeked Puffer Fish (Lagocephalus Sceleratus (Gmelin, 1789)) in Northern Cyprus Sea (Eastern Mediterranean). Toxicon 175, 1–6. FebEpub 2019 Dec 4. PMID: 31833474. 10.1016/j.toxicon.2019.12.002 31833474

[B2] Akyol O. Unal V. Ceyhan T. Bilecenoglu M. (2005). First Confirmed Record of Lagocephalus sceleratus(Gmelin, 1789) in the Mediterranean Sea. J. Fish Biol. 66, 1183–1186. 10.1111/j.0022-1112.2005.00667.x

[B3] Akyol O. Ünal V. (2017). Long Journey of *Lagocephalus Sceleratus* (Gmelin, 1789) throughout the Mediterranean Sea. Nat. Eng. Sci. 2 (3), 41–47. 10.28978/nesciences.369534

[B4] Altschul S. F. Gish W. Miller W. Myers E. W. Lipman D. J. (1990). Basic Local Alignment Search Tool. J. Mol. Biol. 215 (3), 403–410. Oct 5PMID: 2231712. 10.1016/S0022-2836(05)80360-2 2231712

[B5] Andrews S. (2010). FASTQC. A Quality Control Tool for High Throughput Sequence Data.

[B6] Aparicio S. Chapman J. Stupka E. Putnam N. Chia J.-m. Dehal P. (2002). Whole-genome Shotgun Assembly and Analysis of the Genome of *Fugu Rubripes* . Science 297, 1301–1310. 10.1126/science.1072104 12142439

[B7] Arim M. Abades S. R. Neill P. E. Lima M. Marquet P. A. (2006). Spread Dynamics of Invasive Species. Proc. Natl. Acad. Sci. 103, 374–378. 10.1073/pnas.0504272102 16387862 PMC1326152

[B9] Bao Z. Eddy S. R. (2002). Automated De Novo Identification of Repeat Sequence Families in Sequenced Genomes. Genome Res. 12, 1269–1276. 10.1101/gr.88502 12176934 PMC186642

[B10] Bednarska N. G. Wren B. W. Willcocks S. J. (2017). The Importance of the Glycosylation of Antimicrobial Peptides: Natural and Synthetic Approaches. In Drug Discovery Today. Drug Discov. Today 22 (Issue 6), 919–926. 10.1016/j.drudis.2017.02.001 28212948

[B11] Bian L. Li F. Ge J. Wang P. Chang Q. Zhang S. (2019). Chromosome-level Genome Assembly of the Greenfin Horse-Faced Filefish (*Thamnaconus Septentrionalis*) Using Oxford Nanopore PromethION Sequencing and Hi-C Technology. bioRxiv Genomics, 1–25. 10.1101/798744 32390337

[B13] Bolger A. M. Lohse M. Usadel B. (2014). Trimmomatic: A Flexible Trimmer for Illumina Sequence Data. Bioinformatics 30, 2114–2120. 10.1093/bioinformatics/btu170 24695404 PMC4103590

[B14] Bricelj V. M. Connell L. Konoki K. MacQuarrie S. P. Scheuer T. Catterall W. A. (2005). Sodium Channel Mutation Leading to Saxitoxin Resistance in Clams Increases Risk of PSP. Nature 434, 763–767. 10.1038/nature034110.1038/nature03415 15815630

[B15] Castresana J. (2000). Selection of Conserved Blocks from Multiple Alignments for Their Use in Phylogenetic Analysis. Mol. Biol. Evol. 17, 540–552. 10.1093/oxfordjournals.molbev.a026334 10742046

[B16] Chikhi R. Medvedev P. (2014). Informed and Automated K-Mer Size Selection for Genome Assembly. Bioinformatics 30, 31–37. 10.1093/bioinformatics/btt310 23732276

[B17] Chua H. H. Chew L. P. (2009). Puffer Fish Poisoning: a Family Affair. Med. J. Malaysia 64 (2), 181–182. JunPMID: 20058587. 20058587

[B18] Crowl T. A. Crist T. O. Parmenter R. R. Belovsky G. Lugo A. E. (2008). The Spread of Invasive Species and Infectious Disease as Drivers of Ecosystem Change. Front. Ecol. Environ. 6, 238–246. 10.1890/070151

[B19] Daly J. W. (1995). The Chemistry of Poisons in Amphibian Skin. Proc. Natl. Acad. Sci. U S A. 92, 9–13. 10.1073/pnas.92.1.9 7816854 PMC42808

[B20] Darriba D. Posada D. Kozlov A. M. Stamatakis A. Morel B. Flouri T. (2020). ModelTest-NG: A New and Scalable Tool for the Selection of DNA and Protein Evolutionary Models. Mol. Biol. Evol. 37 (Issue 1), 291–294. January. 10.1093/molbev/msz189 31432070 PMC6984357

[B21] De Bie T. Cristianini N. Demuth J. P. Hahn M. W. (2006). CAFE: a Computational Tool for the Study of Gene Family Evolution. Bioinformatics 22, 1269–1271. 10.1093/bioinformatics/btl097 16543274

[B23] Eilbeck K. Moore B. Holt C. Yandell M. (2009). Quantitative Measures for the Management and Comparison of Annotated Genomes. BMC Bioinformatics, 10, 67. 10.1186/1471-2105-10-67 19236712 PMC2653490

[B24] Ellinghaus D. Kurtz S. Willhoeft U. (2008). LTRharvest, an Efficient and Flexible Software for De Novo Detection of LTR Retrotransposons. BMC Bioinformatics 9, 18. 10.1186/1471-2105-9-18 18194517 PMC2253517

[B25] Emms D. M. Kelly S. (2015). OrthoFinder: Solving Fundamental Biases in Whole Genome Comparisons Dramatically Improves Orthogroup Inference Accuracy. Genome Biol. 16, 157. 10.1186/s13059-015-0721-Enright.AJ 26243257 PMC4531804

[B103] Enright A. J. Van Dongen S. Ouzounis C. A. (2002). An Efficient Algorithm For Large-Scale Detection Of Protein Families. Nucleic Acids Res. 30 (7), 1575–1584. 10.1093/nar/30.7.1575 11917018 PMC101833

[B26] Feldman C. R. Brodie E. D. Jr Brodie E. D. 3rd Pfrender M. E. (2012). Constraint Shapes Convergence in Tetrodotoxin-Resistant Sodium Channels of Snakes. Proc. Natl. Acad. Sci. 109 (12), 4556–4561. Mar 20. 10.1073/pnas.1113468109 22392995 PMC3311348

[B27] Filiz H. Er M. (2004). Akdenizin Yeni Misafiri (New Guests in the Mediterranean Sea).

[B29] Flynn J. M. Hubley R. Goubert C. Rosen J. Clark A. G. Feschotte C. (2020). RepeatModeler2 for Automated Genomic Discovery of Transposable Element Families. Proc. Natl. Acad. Sci. USA 117, 9451–9457. 10.1073/pnas.1921046117 32300014 PMC7196820

[B30] Fry B. G. Roelants K. Champagne D. E. Scheib H. Tyndall J. D. A. King G. F. (2009). The Toxicogenomic Multiverse: Convergent Recruitment of Proteins into Animal Venoms. Annu. Rev. Genom. Hum. Genet. 10, 483–511. 10.1146/annurev.genom.9.081307.164356 19640225

[B31] Gao Y. Gao Q. Zhang H. Wang L. Zhang F. Yang C. (2014). Draft Sequencing and Analysis of the Genome of Pufferfish Takifugu Flavidus. DNA Res. 21 (6), 627–637. 10.1093/dnares/dsu025 25053628 PMC4263296

[B33] Geffeney S. L. Williams B. L. Rosenthal J. J. C. Birk M. A. Felkins J. Wisell C. M. (2019). Convergent and Parallel Evolution in a Voltage-Gated Sodium Channel Underlies TTX-Resistance in the Greater Blue-Ringed octopus: *Hapalochlaena Lunulata* . Toxicon 170, 77–84. 10.1016/j.toxicon.2019.09.013 31550451

[B35] Germain R. N. (1994). MHC-dependent Antigen Processing and Peptide Presentation: Providing Ligands for T Lymphocyte Activation. CellElsevier 76 (2), 287–299. 10.1016/0092-8674(94)90336-0 8293464

[B36] Golani D. Appelbaum-Golani B. (2010). *FISH INVASIONS of the MEDITERRANEAN SEA*: Change and Renewal. Sofia Bulgaria: PENSOFT Publishers.

[B37] Golani D. Appelbaum-Golani B. (1977). Guidelines for the Treatment of Animals in Behavioral Research and Teaching. Anim. Behav. 53, 229–234.

[B38] Gurevich A. Saveliev V. Vyahhi N. Tesler G. (2013). QUAST: Quality Assessment Tool for Genome Assemblies. Bioinformatics 29, 1072–1075. 10.1093/bioinformatics/btt086 23422339 PMC3624806

[B39] Halstead B. W. (1988). Poisonous and Venomous Marine Animals of the World: Vertebrates.

[B40] Hanifin C. T. (2010). The Chemical and Evolutionary Ecology of Tetrodotoxin (TTX) Toxicity in Terrestrial Vertebrates. Mar. Drugs 8, 577–593. 10.3390/md8030577 20411116 PMC2857372

[B41] Ho B. Yeo D. S. A. Ding J. L. (1994). A Tetrodotoxin Neutralizing System in the Haemolymph of the Horseshoe Crab, Carcinoscorpius Rotundicauda. Toxicon 32 (7), 755–762. Jul. 10.1016/0041-0101(94)90001-9 7940583

[B42] Holt C. Yandell M. (2011). MAKER2: An Annotation Pipeline and Genome-Database Management Tool for Second-Generation Genome Projects. BMC Bioinformatics 12. 10.1186/1471-2105-12-491 PMC3280279 22192575

[B43] Huerta-Cepas J. Forslund K. Coelho L. P. Szklarczyk D. Jensen L. J. Von Mering C. (2017). Fast Genome-wide Functional Annotation through Orthology Assignment by eggNOG-Mapper. Mol. Biol. Evol. 34, 2115–2122. 10.1093/molbev/msx148 28460117 PMC5850834

[B44] Huerta-Cepas J. Szklarczyk D. Heller D. Hernández-Plaza A. Forslund S. K. Cook H. (2019). EggNOG 5.0: A Hierarchical, Functionally and Phylogenetically Annotated Orthology Resource Based on 5090 Organisms and 2502 Viruses. Nucleic Acids Res. 47, D309–D314. 10.1093/nar/gky1085 30418610 PMC6324079

[B45] Hughes L. C. Ortí G. Huang Y. Sun Y. Baldwin C. C. Thompson A. W. (2018). Comprehensive Phylogeny of ray-finned Fishes (Actinopterygii) Based on Transcriptomic and Genomic Data. Proc. Natl. Acad. Sci. USA 115, 6249–6254. 10.1073/pnas.1719358115 29760103 PMC6004478

[B46] Hughes L. C. Ortí G. Saad H. Li C. White W. T. Baldwin C. C. (2021). Exon Probe Sets and Bioinformatics Pipelines for All Levels of Fish Phylogenomics. Mol. Ecol. Resour. 21, 816–833. 10.1111/1755-0998.13287 33084200

[B47] Jaillon O. Aury J.-M. Brunet F. Petit J.-L. Stange-Thomann N. Mauceli E. (2004). Genome Duplication in the Teleost Fish *Tetraodon nigroviridis* Reveals the Early Vertebrate Proto-Karyotype. Nature 431, 946–957. 10.1038/nature03025 15496914

[B48] Jia L. Zhang J. Ma T. Guo Y. Yu Y. Cui J. (2018). The Function of Fucosylation in Progression of Lung Cancer. Front. Oncol. 8, 565. 10.3389/fonc.2018.00565 30619732 PMC6296341

[B49] Jones P. Binns D. Chang H.-Y. Fraser M. Li W. McAnulla C. (2014). InterProScan 5: Genome-Scale Protein Function Classification. Bioinformatics 30, 1236–1240. 10.1093/bioinformatics/btu031 24451626 PMC3998142

[B50] JostHillis M. C. Hillis D. M. Lu Y. Kyle J. W. Fozzard H. A. Zakon H. H. (2008). Toxin-Resistant Sodium Channels: Parallel Adaptive Evolution across a Complete Gene Family. Mol. Biol. Evol. 25 (Issue 6), 1016–1024. June. 10.1093/molbev/msn025 18258611 PMC2877999

[B51] Kalogirou S. (2013). Ecological Characteristics of the Invasive Pufferfish Lagocephalus Sceleratus (Gmelin, 1789) in the Eastern Mediterranean Sea - a Case Study from Rhodes. Medit. Mar. Sci., 14(2), 251–260. 10.12681/mms.364

[B52] Kaneko Y. Matsumoto G. Hanyu Y. (1997). TTX Resistivity of Na+Channel in Newt Retinal Neuron. Biochem. Biophysical Res. Commun. 240 (3), 651–656. 10.1006/bbrc.1997.7696 9398620

[B53] Kang S. Kim J. H. Jo E. Lee S. J. Jung J. Kim B. M. (2020). Chromosomal‐level Assembly of Takifugu Obscurus (Abe, 1949) Genome Using Third‐generation DNA Sequencing and Hi‐C Analysis. Mol. Ecol. Resour. 20, 520–530. 10.1111/1755-0998.13132 31887246

[B54] Kasapidis P. Peristeraki P. Tserpes G. Magoulas A. (2007). First Record of the Lessepsian Migrant *Lagocephalus Sceleratus* (Gmelin 1789) (Osteichthyes: Tetraodontidae) in the Cretan Sea (Aegean, Greece). Ai 2, 71–73. 10.3391/ai.2007.2.1.9

[B55] Katoh K. Standley D. M. (2013). MAFFT Multiple Sequence Alignment Software Version 7: Improvements in Performance and Usability. Mol. Biol. Evol. 30, 772–780. 10.1093/molbev/mst010 23329690 PMC3603318

[B56] Kawahara R. Miya M. Mabuchi K. Lavoué S. Inoue J. G. Satoh T. P. (2008). Interrelationships of the 11 Gasterosteiform Families (Sticklebacks, Pipefishes, and Their Relatives): A New Perspective Based on Whole Mitogenome Sequences from 75 Higher Teleosts. Mol. Phylogenet. Evol. 46, 224–236. 10.1016/j.ympev.2007.07.009 17709262

[B57] Kielbasa S. M. Wan R. Sato K. Horton P. Frith M. C. (2011). Adaptive Seeds Tame Genomic Sequence Comparison. Genome Res. 21 (3), 487PMC3044862–493. MarEpub 2011 Jan 5. PMID: 21209072; PMCID. 10.1101/gr.113985.110 21209072 PMC3044862

[B58] Kim D. Langmead B. Salzberg S. L. (2015). HISAT: A Fast Spliced Aligner with Low Memory Requirements. Nat. Methods 12, 357–360. 10.1038/nmeth.3317 25751142 PMC4655817

[B59] Kolar C. S. Lodge D. M. (2001). Progress in Invasion Biology. 10.1016/s0169-5347(01)02101-2 11245943

[B60] Kolmogorov M. Yuan J. Lin Y. Pevzner P. A. (2019). Assembly of Long, Error-Prone Reads Using Repeat Graphs. Nat. Biotechnol. 37, 540–546. 10.1038/s41587-019-0072-8 30936562

[B61] Korf I. (2004). Gene Finding in Novel Genomes. BMC Bioinformatics 5, 59. 10.1186/1471-2105-5-59 15144565 PMC421630

[B62] Kosker A. R. Özogul F. Durmus M. Ucar Y. Ayas D. Regenstein J. M. (2016). Tetrodotoxin Levels in Pufferfish (Lagocephalus Sceleratus) Caught in the Northeastern Mediterranean Sea. Food Chem. 210, 332–337. Nov 1Epub 2016 Apr 27. PMID: 27211655. 10.1016/j.foodchem.2016.04.122 27211655

[B63] Kozlov A. M. Darriba D. Flouri T. Morel B. Stamatakis A. RAxML-N. G. (2019). RAxML-NG: a Fast, Scalable and User-Friendly Tool for Maximum Likelihood Phylogenetic Inference. Bioinformatics 35 (Issue 21), 4453–4455. 1 November. 10.1093/bioinformatics/btz305 31070718 PMC6821337

[B64] Krzywinski M. Schein J. Birol I. Connors J. Gascoyne R. Horsman D. (2009). Circos: an Information Aesthetic for Comparative Genomics. Genome Res. 19 (9), 1639–1645. SepEpub 2009 Jun 18. PMID: 19541911; PMCID: PMC275213. 10.1101/gr.092759.109 19541911 PMC2752132

[B65] Lee K. A. Klasing K. C. (2004). A Role for Immunology in Invasion Biology. Trends Ecol. Evol. 19, 523–529. 10.1016/j.tree.2004.07.012 16701317

[B67] Li H. (2018). Minimap2: Pairwise Alignment for Nucleotide Sequences. Bioinformatics 34, 3094–3100. 10.1093/bioinformatics/bty191 29750242 PMC6137996

[B68] Li W. Godzik A. (2006). Cd-hit: a Fast Program for Clustering and Comparing Large Sets of Protein or Nucleotide Sequences. Bioinformatics 22, 1658–1659. 10.1093/bioinformatics/btl158 16731699

[B69] Malý P. Thall A. D. Petryniak B. Rogers C. E. Smith P. L. Marks R. M. (1996). The α(1,3)Fucosyltransferase Fuc-TVII Controls Leukocyte Trafficking through an Essential Role in L-, E-, and P-Selectin Ligand Biosynthesis. Cell 86, 643–653. 10.1016/s0092-8674(00)80137-3 8752218

[B71] Meynard C. N. Mouillot D. Mouquet N. Douzery E. J. P. (2012). A Phylogenetic Perspective on the Evolution of Mediterranean Teleost Fishes. PLoS ONE 7, e36443. 10.1371/journal.pone.0036443 22590545 PMC3348158

[B72] Nagashima Y. Arakawa O. (2016). “Pufferfish Poisoning and Tetrodotoxin,” in Marine and Freshwater Toxins. Editors Gopalakrishnakone P. HaddadJr. V. Tubaro A. Kim E. Kem W., and, 259–284. 10.1007/978-94-007-6419-410.1007/978-94-007-6419-4_12

[B74] Natsidis P. Tsakogiannis A. Pavlidis P. Tsigenopoulos C. S. Manousaki T. (2019). Phylogenomics Investigation of Sparids (Teleostei: Spariformes) Using High-Quality Proteomes Highlights the Importance of Taxon Sampling. Commun. Biol. 2, 1–10. 10.1038/s42003-019-0654-5 31701028 PMC6825128

[B80] Ou S. Jiang N. (2018). LTR_retriever: A Highly Accurate and Sensitive Program for Identification of Long Terminal Repeat Retrotransposons. Plant Physiol. 176, 1410–1422. 10.1104/pp.17.01310 29233850 PMC5813529

[B81] Palumbi S. R. (2001). The Evolution Explosion: How Humans Cause Rapid Evolutionary Change. New York: W. W. Norton.

[B82] Pan H. Yu H. Ravi V. Li C. Lee A. P. Lian M. M. (2016). The Genome of the Largest Bony Fish, Ocean Sunfish (*Mola mola*), Provides Insights into its Fast Growth Rate. GigaSci 5, 36. 10.1186/s13742-016-0144-3 PMC5016917 27609345

[B83] Pertea M. Pertea G. M. Antonescu C. M. Chang T.-C. Mendell J. T. Salzberg S. L. (2015). StringTie Enables Improved Reconstruction of a Transcriptome from RNA-Seq Reads. Nat. Biotechnol. 33, 290–295. 10.1038/nbt.3122 25690850 PMC4643835

[B85] Por F. D. (1971). One Hundred Years of Suez Canal-A Century of Lessepsian Migration: Retrospect and Viewpoints. Syst. Zoolog. 20, 138–159. 10.2307/2412054

[B86] Price A. L. Jones N. C. Pevzner P. A. (2005). De Novo identification of Repeat Families in Large Genomes. Bioinformatics 21, i351–i358. 10.1093/bioinformatics/bti1018 15961478

[B87] Ranallo-Benavidez T. R. Jaron K. S. Schatz M. C. (2020). GenomeScope 2.0 and Smudgeplot for Reference-free Profiling of Polyploid Genomes. Nat. Commun. 11, 1432. 10.1038/s41467-020-14998-3 32188846 PMC7080791

[B89] Sanderson M. J. (2003). r8s: Inferring Absolute Rates of Molecular Evolution and Divergence Times in the Absence of a Molecular Clock. Bioinformatics 19 (Issue 2), 301–302. 22 January. 10.1093/bioinformatics/19.2.301 12538260

[B91] Satin J. Kyle J. W. Chen M. Bell P. Cribbs L. L. Fozzard H. A. (1992). A Mutant of TTX-Resistant Cardiac Sodium Channels with TTX-Sensitive Properties. Science 256, 1202–1205. 10.1126/science.256.5060.1202 1375397

[B92] Sax D. Stachowicz J. Brown J. Bruno J. Dawson M. Gaines S. (2007). Ecological and Evolutionary Insights from Species Invasions. Trends Ecol. Evol. 22, 465–471. 10.1016/j.tree.2007.06.009 17640765

[B93] Shao F. Han M. Peng Z. (2019). Evolution and Diversity of Transposable Elements in Fish Genomes. Sci. Rep. 9. 10.1038/s41598-019-51888-1 PMC6817897 31659260

[B94] Shao F. Wang J. Xu H. Peng Z. (2018). FishTEDB: a Collective Database of Transposable Elements Identified in the Complete Genomes of Fish. Database 2018 (2018), 106. 10.1093/database/bax106 PMC6404401 29688350

[B95] Simão F. A. Waterhouse R. M. Ioannidis P. Kriventseva E. V. Zdobnov E. M. (2015). BUSCO: Assessing Genome Assembly and Annotation Completeness with Single-Copy Orthologs. Bioinformatics 31, 3210–3212. 10.1093/bioinformatics/btv351 26059717

[B97] Soong T. Venkatesh B. (2006). Adaptive Evolution of Tetrodotoxin Resistance in Animals. Trends Genet. 22 (11), 621–626. NovEpub 2006 Sep 7. 10.1016/j.tig.2006.08.010 16959367

[B98] Stanke M. Keller O. Gunduz I. Hayes A. Waack S. Morgenstern B. (2006). AUGUSTUS: Ab Initio Prediction of Alternative Transcripts. Nucleic Acids Res. 34, W435–W439. Jul 1(Web Server issue)PMCID: PMC1538822.4. 10.1093/nar/gkl200.PMID:16845043 16845043 PMC1538822

[B99] Stapley J. Santure A. W. Dennis S. R. (2015). Transposable Elements as Agents of Rapid Adaptation May Explain the Genetic Paradox of Invasive Species. Mol. Ecol. 24, 2241–2252. 10.1111/mec.13089 25611725

[B100] Stein C. Caccamo M. Laird G. Leptin M. (2007). Conservation and Divergence of Gene Families Encoding Components of Innate Immune Response Systems in Zebrafish. Genome Biol. 8, R251. 10.1186/gb-2007-8-11-r251 18039395 PMC2258186

[B102] Tarailo‐Graovac M. Chen N. (2009). Using RepeatMasker to Identify Repetitive Elements in Genomic Sequences. Curr. Protoc. Bioinformatics 25. 4 (Issue SUPPL. 25). 10.1002/0471250953.bi0410s25 19274634

[B104] Vaser R. Šikić M. (2020). Raven: A De Novo Genome Assembler for Long Reads. bioRxiv, 2020. 10.1101/2020.08.07.242461

[B105] Vaser R. Sović I. Nagarajan N. Šikić M. (2017). Fast and Accurate De Novo Genome Assembly from Long Uncorrected Reads. Genome Res. 27, 737–746. 10.1101/gr.214270.116 28100585 PMC5411768

[B106] Venkatesh B. Lu S. Q. Dandona N. See S. L. Brenner S. Soong T. W. (2005). Genetic Basis of Tetrodotoxin Resistance in Pufferfishes. Curr. Biol. 15, 2069–2072. 10.1016/j.cub.2005.10.068 16303569

[B107] Walker B. J. Abeel T. Shea T. Priest M. Abouelliel A. Sakthikumar S. (2014). Pilon: An Integrated Tool for Comprehensive Microbial Variant Detection and Genome Assembly Improvement. PLoS One 9, e112963. 10.1371/journal.pone.0112963 25409509 PMC4237348

[B108] Waterhouse A. Procter J. Martin D. A. Barton G. J. (2005). Jalview: Visualization and Analysis of Molecular Sequences, Alignments, and Structures. BMC Bioinformatics 6, P28. 10.1186/1471-2105-6-S3-P28

[B109] Wheeler T. J. (2009). Large-scale Neighbor-Joining with NINJA. Lecture Notes in Computer Science (Including Subseries Lecture Notes in Artificial Intelligence and Lecture Notes. Bioinformatics 5724 LNBI, 375–389. 10.1007/978-3-642-04241-6_31

[B110] Whitelaw B. L. CookeCooke I. R. Finn J. da Fonseca R. R. Ritschard E. A. Gilbert M. T. P. (2020). Adaptive Venom Evolution and Toxicity in Octopods Is Driven by Extensive Novel Gene Formation, Expansion, and Loss. GigaScience 9 (Issue). 11, Novembergiaa120. 10.1093/gigascience/giaa120 PMC7656900 33175168

[B113] Yamanoue Y. Miya M. Doi H. Mabuchi K. Sakai H. Nishida M. (2011). Multiple Invasions into Freshwater by Pufferfishes (Teleostei: Tetraodontidae): A Mitogenomic Perspective. PLoS ONE 6, e17410. 10.1371/journal.pone.0017410 21364898 PMC3045446

[B115] Yotsu-Yamashita M. Nishimori K. Nitanai Y. Isemura M. Sugimoto A. Yasumoto T. (2000). Binding Properties of 3H-PbTx-3 and 3H-Saxitoxin to Brain Membranes and to Skeletal Muscle Membranes of Puffer Fish Fugu Pardalis and the Primary Structure of a Voltage-Gated Na+ Channel α-Subunit (fMNa1) from Skeletal Muscle of *F. pardalis* . Biochem. Biophysical Res. Commun. 267 (1), 403–412. Jan 7. 10.1006/bbrc.1999.1974 10623632

[B116] Yu F. H. Yarov-Yarovoy V. Gutman G. A. Catterall W. A. (2005). Overview of Molecular Relationships in the Voltage-Gated Ion Channel Superfamily. Pharmacol. Rev. 57, 387–395. 10.1124/pr.57.4.13 16382097

[B117] Yuan Z. Liu S. Zhou T. Tian C. Bao L. Dunham R. (2018). Comparative Genome Analysis of 52 Fish Species Suggests Differential Associations of Repetitive Elements with Their Living Aquatic Environments. BMC Genomics 19. 10.1186/s12864-018-4516-1 PMC5811955 29439662

[B118] Zenetos Α. Gofas S. Morri C. Rosso A. Violanti D. Garcia Raso J. E. (2012). Alien Species in the Mediterranean Sea by 2012. A Contribution to the Application of European Union's Marine Strategy Framework Directive (MSFD). Part 2. Introduction Trends and Pathways. Medit. Mar. Sci. 13, 328. 10.12681/mms.327

[B119] Zafeiropoulos H. Gioti A. Ninidakis S. PotirakisParagkamian A. Paragkamian S. Angelova N. (2021). 0s and 1s in marine Molecular Research: a Regional HPC Perspective. GigaScience 10 (Issue 8), giab053. August. 10.1093/gigascience/giab053 34405237 PMC8371273

[B120] Zakon H. H. Jost M. C. Lu Y. (2011). Expansion of Voltage-dependent Na+ Channel Gene Family in Early Tetrapods Coincided with the Emergence of Terrestriality and Increased Brain Complexity. Mol. Biol. Evol. 28, 1415–1424. 10.1093/molbev/msq325 21148285 PMC3058772

[B122] Zhou Y. Xiao S. Lin G. Chen D. Cen W. Xue T. (2019a). Chromosome Genome Assembly and Annotation of the Yellowbelly Pufferfish with PacBio and Hi-C Sequencing Data. Sci. Data 6, 267. 10.1038/s41597-019-0279-z 31704938 PMC6841922

[B123] Zhou Z. Liu B. Chen B. Shi Y. Pu F. Bai H. (2019b). The Sequence and De Novo Assembly of *Takifugu Bimaculatus* Genome Using PacBio and Hi-C Technologies. Sci. Data 6, 187. 10.1038/s41597-019-0195-2 31570724 PMC6768875

[B124] Zou S. (2020). Genetic Patterns for Adaptive Evolution of TTX in Nassarius. Authorea August 12, 2020. 10.22541/au.159724546.6127140

