# Supplementary material for: Genome Analysis of Lagocephalus sceleratus: Unraveling the Genomic Landscape of a Successful Invader
Source: Front Genet. 2021 Dec 8;12:790850. doi: 10.3389/fgene.2021.790850 (PMC8692874; doi:10.3389/fgene.2021.790850)
Supplement: Supplementary file 1 [file DataSheet1.docx]

**Supplementary Table 1**. *L. sceleratus* specific genes from the 2 rapidly expanded gene families.

| **Entry** | **Protein names** | **Gene names** | **Organism** |
| --- | --- | --- | --- |
| Q4RTI4 | Chromosome 1 SCAF14998 | GSTENG00029235001 | *Tetraodon nigroviridis* |
| A0A3P9KVW8 | Uncharacterized protein | - | *Oryzias latipes* |
| A0A3B4VFG6 | Reverse transcriptase domain-containing protein | - | *Seriola dumerili* |
| A0A0G2L2B6 | Reverse transcriptase domain-containing protein | - | - |
| A0A3B5QQH6 | Uncharacterized protein | - | *Xiphophorus maculatus* |

**Supplementary Table 2**. Summary of whole genome synteny plots. The last row contains the *L. sceleratus* contigs that are represented within the chromosomes of the other puffers.

| **Species** | **Chromosomes** | | | | | | | | | | | | | | | | | | | | | |
| --- | --- | --- | --- | --- | --- | --- | --- | --- | --- | --- | --- | --- | --- | --- | --- | --- | --- | --- | --- | --- | --- | --- |
| *T.bimaculatus* | 1 | 2 | 3 | 4 | 5 | 6 | 7 | 8 | 9 | 10 | 11 | 12 | 13 | 14 | 15 | 16 | 17 | 18 | 19 | 20 | 21 | 22 |
| *T.flavidus* | 1 | 3 | 2 | 5 | 4 | 7 | 8 | 6 | 14 | 10 | 12 | 13 | 11 | 9 | 15 | 18 | 17 | 16 | 19 | - | 20 | 22 |
| *T.rubripes* | 1 | 2 | 13 | 21 | 19 | 20 | 3 | 17 | 15 | 7 | 11 | 9 | 17 | 14 | 22 | 5 | 10 | 2 | 16 | - | 6 | 18 |
| *T.nigroviridis* | 2 | - | 5 | 12 | 11 | 1 | 9 | 18 | 7 | 8 | 16 | 13 | 4 | 20 | 15 | - | 6 | 10 | 12 | 10 | 4 | 19 |
| Contigs | 1, 14 | 6, 2 | 4, 38 | 7 | 22,33,34 | 20, 39 | 8,35, 36 | 1,2 24 | 18, 26 | 19, 31 | 10, 32 | 23, 27 | 3, 29 | 13, 37 | 11 | 9 | 2, 40 | 16 | 6 | 17, 18 | 12, 25 | 15, 30 |

**Supplementary Table 3.** Species included in the phylogenomic analysis.

| **Species** | **Series (for Percomorphaceae)** | **Source** | **Reference** | **#of proteins** |
| --- | --- | --- | --- | --- |
| *A. mexicanus* | (Ostariophysi) | Ensembl database | McGaugh, S. E. et al., 2014 | 22,998 |
| *B. pectinirostris* | Gobiaria | NCBI ftp server | You, X. et al., 2014 | 21,541 |
| *C. argus* | Anabantaria | GigaDB | Xu, J. et al., 2017 | 20,541 |
| *C. semilaevis* | Carangaria | NCBI ftp server | Chen, S. et al., 2014 | 24,489 |
| *D. labrax* | Eupercaria | species database | Tine, M. et al., 2014 | 26,719 |
| *D. rerio* | (Ostariophysi) | Ensembl database | Howe, K. et al., 2013 | 25,644 |
| *G. aculeatus* | Eupercaria | Ensembl database | Jones, F. C. et al., 2012 | 20,625 |
| *G. morhua* | (Paracanthopterygii) | Ensembl database | Star, B. et al., 2011 | 19,978 |
| *H. erectus* | Syngnatharia | GigaDB | Lin, Q. et al., 2017 | 20,788 |
| *K. marmoratus* | Ovalentaria | NCBI ftp server | Kelley, J. L. et al., 2016 | 25,257 |
| *L. calcarifer* | Carangaria | NCBI ftp server | Vij, S. et al., 2016 | 22,221 |
| *L. crocea* | Eupercaria | NCBI ftp server | Ao, J. et al., 2015 | 28,009 |
| *L. oculatus* | (Holostei) | Ensembl database | Braasch, I. et al., 2016 | 18,304 |
| *L. sceleratus* | Eupercaria | in-house sequenced | Current study | 21,251 |
| *M. albus* | Anabantaria | NCBI ftp server | Yi, M. et al., 2014 | 24,943 |
| *M. mola* | Eupercaria | GigaDB | Pan, H. et al., 2016 | 19,605 |
| *M. peelii* | Eupercaria | GigaDB | Austin, C. M. et al., 2017 | 26,539 |
| *N. coriiceps* | Eupercaria | NCBI ftp server | Shin, S. C. et al., 2014 | 25,937 |
| *O. latipes* | Ovalentaria | Ensembl database | Kasahara, M. et al. 2007 | 19,603 |
| *O. niloticus* | Ovalentaria | Ensembl database | Brawand, D. et al. 2014 | 21,383 |
| *P. charcoti* | Eupercaria | provided by authors | Ahn, D. H. et al., 2017 | 32,713 |
| *P. formosa* | Ovalentaria | Ensembl database | Warren, W. C. et al., 2018 | 23,315 |
| *S. aurata* | Eupercaria | in-house sequenced | Pauletto, M. et al., 2018 | 61,850 |
| *S. dumerili* | Carangaria | NCBI ftp server | Araki et al., unpublished | 24,000 |
| *T. bimaculatus* | Eupercaria | Uniprot ftp server | Zhou, Z., et al., 2019b | 19,334 |
| *T. flavidus* | Eupercaria | Uniprot ftp server | Gao et al., 2014 | 29,076 |
| *T. nigroviridis* | Eupercaria | Ensembl database | Jatllon, O. et al., 2004 | 19,511 |
| *T. rubripes* | Eupercaria | Ensembl database | Aparicio, S. et al., 2002 | 18,433 |
| *T. thynnus* | Pelagiaria | species database | Nakamura, Y. et al., 2013 | 26,433 |
| *X. maculatus* | Ovalentaria | Ensembl database | Schartl, M. et al., 2013 | 20,343 |

**Supplementary Table 4.** Number of genes that are involved in rapidly expanded gene families and its functionality.

| **Function** | **# Of genes** |
| --- | --- |
| Transposase | 24 |
| Fucosyltransferase 9 (alpha (1,3) fucosyltransferase) | 23 |
| ENV polyprotein (coat polyprotein) | 21 |
| K02A2.6-like | 12 |
| SCAN domain | 12 |
| B-cell receptor CD22-like | 7 |
| Reverse transcriptase (RNA-dependent DNA polymerase) | 6 |
| Podospora anserina S mat genomic DNA chromosome | 6 |
| Ig heavy chain Mem5-like | 6 |
| Si ch211-286b4.4 | 6 |
| Nuclear migration along micro-filament | 5 |
| Si ch211-81n22.1 | 5 |
| Immunoglobulin C-Type | 5 |
| Immunoglobulin V-Type | 5 |
| Fish-specific NACHT associated domain | 5 |
| DDE superfamily endonuclease | 4 |
| Receptor | 4 |
| protein dimerization activity | 3 |
| Immunoglobulin V-set domain | 3 |
| cytoskeletal anchoring at nuclear membrane | 3 |
| Early B-cell factor 3 | 3 |
| Early B-cell factor 1 | 2 |
| Early B-cell factor 2 | 2 |
| Sad1 and UNC84 domain containing 1 | 2 |
| Serpentine type 7TM GPCR chemoreceptor Srx | 2 |
| Fucosyltransferase 7 (alpha (1,3) fucosyltransferase) | 2 |
| Immunoglobulin C1-set domain | 2 |
| Retrotransposable element Tf2 155 kDa protein type 1-like | 2 |
| Belongs to the MHC class I family | 2 |
| Early B-cell factor | 2 |
| Solute carrier family 9, subfamily A (NHE3, cation proton antiporter 3), member 3 | 1 |
| Coreceptor activity involved in Wnt signaling pathway, planar cell polarity pathway | 1 |
| Phosphodiesterase 4D | 1 |
| B-cell receptor | 1 |
| Interleukin 4 induced 1 | 1 |
| Metal dependent phosphohydrolase with conserved ‘HD’ motif. | 1 |
| ST3 beta-galactoside alpha-2,3-sialyltransferase 1 | 1 |
| N-acetyltransferase | 1 |
| Reverse transcriptase | 1 |
| Galactosyltransferase | 1 |
| Transposition, RNA-mediated | 1 |
| DNA-binding transcription factor activity, RNA polymerase II-specific | 1 |
| Endonuclease/Exonuclease/phosphatase family | 1 |
| Phosphodiesterase 4A, cAMP-specific | 1 |
| Receptor accessory protein-like 2 | 1 |
| Monovalent cation proton antiporter 1 (CPA1) transporter (TC 2.A.36) family | 1 |
| Si dkey-24p1.1 | 1 |
| Calcium binding protein | 1 |
| Pao retrotransposon peptidase | 1 |
| SH3 domain binding kinase family, member 2 | 1 |
| Ribonuclease H protein | 1 |
| Purinergic receptor P2Y, G-protein coupled, 12 | 1 |
| Fucosyltransferase 4 (alpha (1,3) fucosyltransferase, myeloid-specific) | 1 |
| Calcium ion binding | 1 |

**Supplementary Table 5.** Number of genes that are involved in rapidly contracted gene families and its functionality.

| **Function** | **# Of genes** |
| --- | --- |
| Glutamate receptor, ionotropic | 7 |
| regulator of G-protein signaling | 7 |
| protein heterodimerization activity | 6 |
| Belongs to the beta gamma-crystallin family | 6 |
| ryanodine receptor | 5 |
| Belongs to the G-protein coupled receptor 1 family | 3 |
| Beta/gamma crystallins | 3 |
| 7 transmembrane receptors (rhodopsin family) | 2 |
| Opsin 6, group member a | 2 |
| fibulin-like extracellular matrix protein | 2 |
| Proprotein convertase subtilisin kexin type | 2 |
| Belongs to the G-protein coupled receptor 1 family. Opsin subfamily | 2 |
| Teleost multiple tissue opsin | 2 |
| Glutamyl aminopeptidase | 1 |
| Opsin 8, group member c | 1 |
| Aminopeptidase puromycin sensitive | 1 |
| C-terminus of histone H2A | 1 |
| Endoplasmic reticulum aminopeptidase 1 | 1 |
| Opsin 4xb | 1 |
| Regulator of G-protein signaling 3-like | 1 |
| Subtilase family | 1 |
| Serine-type endopeptidase activity | 1 |
| regulator of G-protein | 1 |
| Histone H2A | 1 |
| aminopeptidase | 1 |
| Ryanodine Receptor TM 4-6 | 1 |
| Opsin 4a (melanopsin) | 1 |
| Rhodopsin | 1 |
| Fibulin 2 | 1 |
| Protein-chromophore linkage | 1 |
| Green-sensitive opsin-like | 1 |
| Opsin 1 (cone pigments), long-wave-sensitive | 1 |
| H2A histone family, member | 1 |
| H2A histone family, member Y2 | 1 |
| Incorporated into fibronectin-containing matrix fibers. May play a role in cell adhesion and migration along protein fibers within the extracellular matrix (ECM). Could be important for certain developmental processes and contribute to the supra-molecular organization of ECM architecture, to those of basement membranes | 1 |
| Vertebrate ancient long opsin | 1 |
| Peptidase family M1 domain | 1 |
| Ataxin 10 | 1 |
| Transcription intermediary factor 1-alpha-like | 1 |
| Belongs to the histone H2B family | 1 |
| negative regulation of adenylate cyclase-inhibiting adrenergic receptor signaling pathway involved in heart process | 1 |
| Belongs to the protein kinase superfamily. Tyr protein kinase family | 1 |
| Opsin 4.1 | 1 |
| H2A histone family, member Y | 1 |
| Si ch211-196h16.12 | 1 |
| Histone H1 like | 1 |
| Leucyl cystinyl aminopeptidase | 1 |
| Gamma-crystallin M2-like | 1 |
| Histone H2A-like | 1 |
| Belongs to the histone H2A family | 1 |


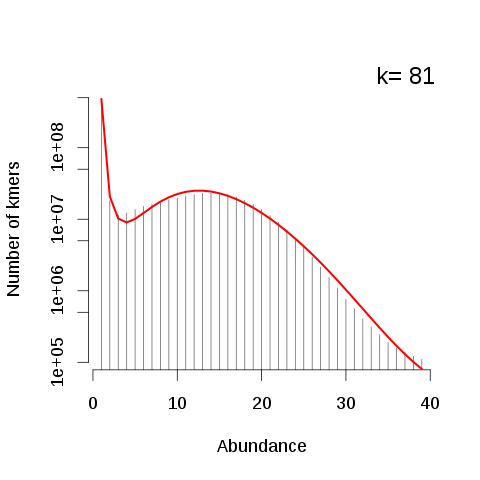

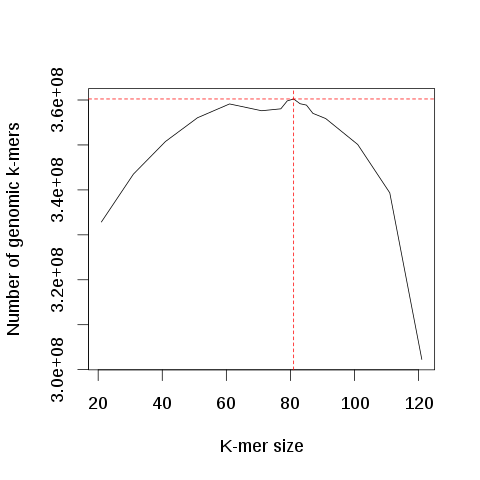


**Supplementary Figure 1.** Kmergenie results on *L. sceleratus* illumina data


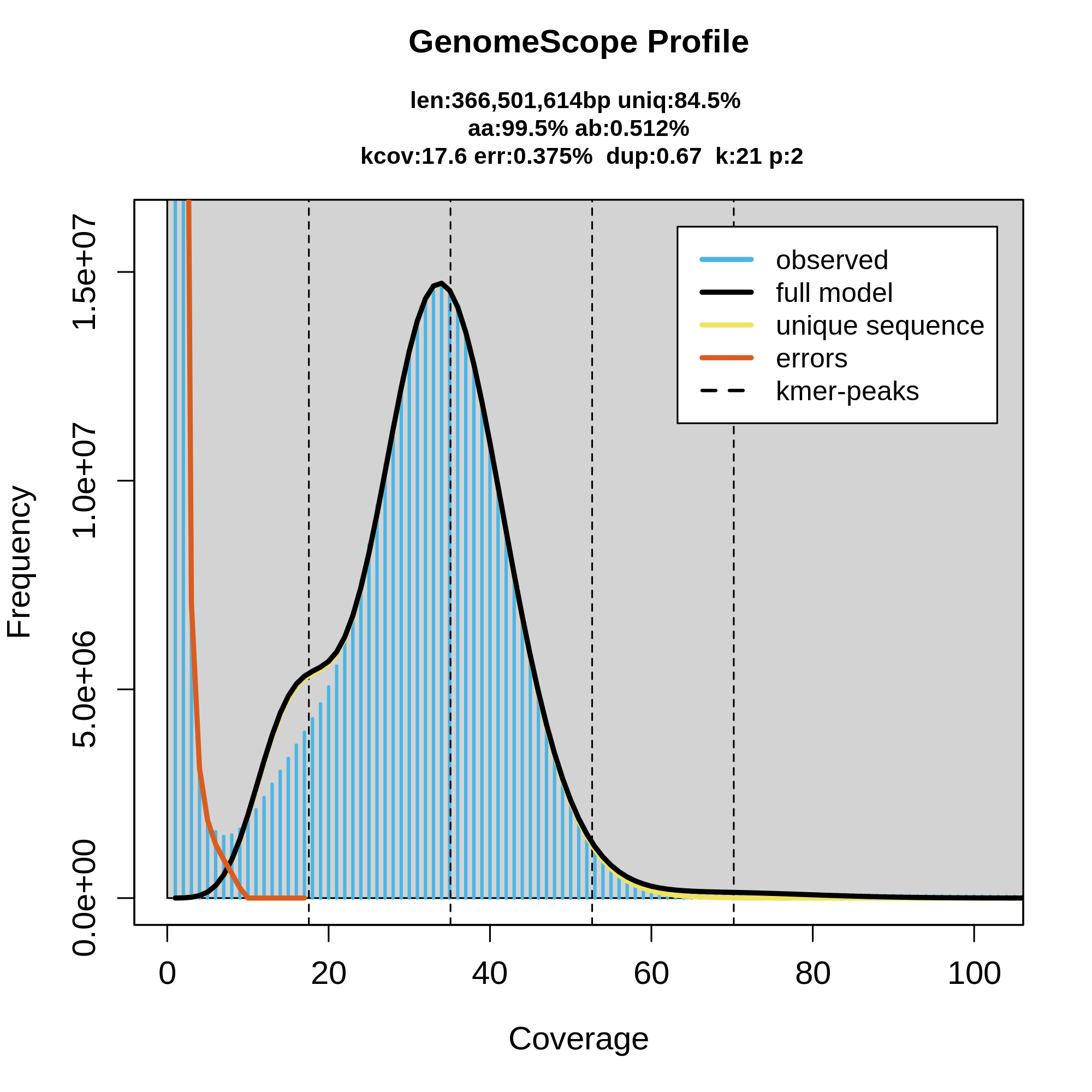


**Supplementary Figure 2.** Genomescope2 kmer frequency profile for *L. sceleratus* genome assembly.
